# Simulation of Molecular Dynamics of SARS-CoV-2 S-Protein in the Presence of Multiple Arbidol Molecules: Interactions and Binding Mode Insights

**DOI:** 10.3390/v14010119

**Published:** 2022-01-10

**Authors:** Sophia S. Borisevich, Edward M. Khamitov, Maxim A. Gureev, Olga I. Yarovaya, Nadezhda B. Rudometova, Anastasiya V. Zybkina, Ekaterina D. Mordvinova, Dmitriy N. Shcherbakov, Rinat A. Maksyutov, Nariman F. Salakhutdinov

**Affiliations:** 1 Ufa Federal Research Center, Laboratory of Chemical Physics, Ufa Institute of Chemistry, RAS, Octyabrya pr., 71, 450054 Ufa, Russia; khamitovem@gmail.com; 2 Research Center “Digital Biodesign and Personalized Healthcare”, I.M. Sechenov First Moscow State Medical University, 119991 Moscow, Russia; max_technik@mail.ru; 3 Department of Computational Biology, Sirius University of Science and Technology, 354349 Sochi, Russia; 4 Department of Medicinal Chemistry, N.N. Vorozhtsov Novosibirsk Institute of Organic Chemistry SB RAS, Lavrent’ev Av., 630090 Novosibirsk, Russia; mordvinova97@mail.ru (E.D.M.); anvar@nioch.nsc.ru (N.F.S.); 5 State Research Center of Virology and Biotechnology VECTOR, Rospotrebnadzor, Koltsovo, 630559 Novosibirsk, Russia; andreeva_nb@vector.nsc.ru (N.B.R.); zybkina_av@vector.nsc.ru (A.V.Z.); dnshcherbakov@gmail.com (D.N.S.); maksyutov_ra@vector.nsc.ru (R.A.M.)

**Keywords:** SARS-CoV-2, coronavirus surface protein S-spike, arbidol, molecular dynamics, molecular docking, pseudoviral system

## Abstract

In this work, we evaluated the antiviral activity of Arbidol (Umifenovir) against SARS-CoV-2 using a pseudoviral system with the glycoprotein S of the SARS-CoV-2 virus on its surface. In order to search for binding sites to protein S of the virus, we described alternative binding sites of Arbidol in RBD and in the ACE-2-RBD complex. As a result of our molecular dynamics simulations combined with molecular docking data, we note the following fact: wherever the molecules of Arbidol bind, the interaction of the latter affects the structural flexibility of the protein. This interaction may result both in a change in the shape of the domain–enzyme binding interface and simply in a change in the structural flexibility of the domain, which can subsequently affect its affinity to the enzyme. In addition, we examined the possibility of Arbidol binding in the stem part of the surface protein. The possibility of Arbidol binding in different parts of the protein is not excluded. This may explain the antiviral activity of Arbidol. Our results could be useful for researchers searching for effective SARS-CoV-2 virus inhibitors targeting the viral entry stage.

## 1. Introduction

On 11 March 2020, “deeply concerned both by the alarming levels of spread and severity, and by the alarming levels of inaction, the WHO (World Health Ogranization) made the assessment that COVID-19 could be characterized as a pandemic” [1]. More than a year has passed, yet the pandemic continues around the world, and there is no country in which severe acute respiratory syndrome caused by SARS-CoV-2 coronavirus has not occurred. Meanwhile, coronaviruses, RNA viruses of the Coronaviridae family, are widespread in nature and affect both mammals and birds. Coronavirus HCoV, which affects humans, was first isolated in 1965 in humans suffering from cold symptoms but did not attract much scientific attention. However, at the beginning of the 21st century, new members of the coronavirus group that can cause severe pneumonia appeared: SARS-CoV (severe acute respiratory coronavirus syndrome), MERS-CoV (Middle East respiratory coronavirus syndrome) and, finally, a new type of SARS-CoV-2 (or COVID-19). SARS-CoV-2 was identified in December 2019 in China [2]. Diseases caused by these coronaviruses have similar clinical features, whereas SARS-CoV-2 is characterized by high virulence and aggressiveness as well as lower lethality [3]. According to [4], SARS-CoV-2 has estimated mortality of 2.3%, which is lower than SARS-CoV (9.5%) and MERS-CoV (34.4%).

The SARS-CoV-2 virus poses a significant threat not only to the lives and to the health of the global population, but also negatively affects the world economy. International efforts to suppress the ongoing pandemic are significant [5,6]. There are several strategies for controlling human viral diseases. The first is disease prevention, which includes vaccination and a set of sanitary and epidemiological measures. The process of developing vaccines usually takes a long time, about 10–15 years. With the ongoing pandemic, however, this timeframe has been shortened to 12–18 months [7]. A concerted global effort by both private and government organizations has led to several SARS-CoV-2 vaccine candidates moving into Phase III and Phase IV clinical trials [8,9,10,11]. A number of countries have approved the use of vaccines for mass vaccination. Most countries in the world are already vaccinating their populations, which gives us hope for a shorter pandemic. However, the example of widespread vaccination of people against the influenza virus shows that there is always some group of patients for whom vaccination is not possible due to several medical contraindications [12]. An alternative to prophylactic vaccination is the use of specific antiviral drugs.

To develop a search strategy for potential antiviral drugs, it is necessary to understand the structure of the virus and its life cycle. The coronavirus virion, covered with a lipid shell, has a spheroid shape, and is 80–200 nm in size. The viral capsid contains a nucleocapsid (ribonucleoprotein) that contains the viral genome. Generally, coronaviruses consist of a canonical set of four major structural proteins: the glycoprotein or surface spike proteins (further S-protein), matrix (M) and envelope (E) proteins, each located in the lipid shell of the virus, and the nucleocapsid protein (N), which forms a complex with the virus genetic material. Theoretically, each stage of the virus life cycle could be a potential target for drug therapy.

**Figure 1:**
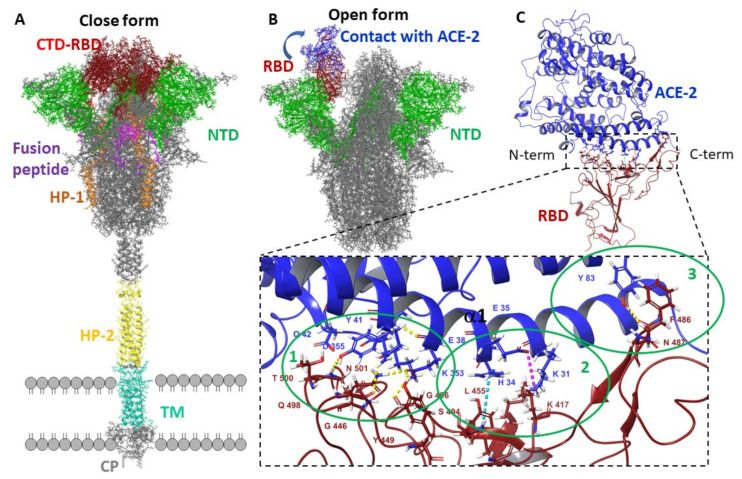
Molecular structure of SARS-CoV-2 coronavirus surface protein: (**A**)—closed conformation corresponds to PDB [19] code 6VXX [2]: CTD—C-terminal domain amino acids are shown in red, receptor-binding domain (RBD) amino acids are shown in dark red; NTD–N-terminal domain is shown in green; amino acids of the fusion peptide are shown in purple; the first (HR1) and second (HR-2) heptad repeats are shown in orange and yellow, respectively; the trans-membrane (TM) domain is highlighted in turquoise; SR is the cytoplasmic tail. (**B**)—Protein activation is associated with the event of open conformation formation, where amino acids of the RBD domain entering the contact region with the ACE-2 enzyme become available for interaction (B—highlighted in blue PBD code 6VSB [2]). (**C**)—RDB-ACE-2 complex (PDB code 6VW1 [18]): amino acids forming a series of intermolecular contacts are shown: hydrogen and salt bridges are shown with yellow and purple dashed lines; π–π stacking contacts are shown with a blue dashed line.

The surface protein CoV-S plays a crucial role in the viral life cycle: it regulates entry into the host cell and is the main target for the host humoral immune response. In the viral membrane, the protein is involved in two important events: binding to the cell receptor and subsequent fusion of the viral and cell membranes. Surface glycoprotein S is a type I transmembrane fusion protein, weighing 180–200 kDa. The N-end of the protein faces the extracellular space and is retained in the viral membrane through a transmembrane domain with a short C-terminal segment facing the intracellular space. Figure 1A shows the atomic-molecular structure of the glycoprotein in a closed conformation with different domains highlighted in color. Structural modeling of the protein monomers of coronavirus S shows that subunits S1 and S2 form the “bulb” head and “stem” region, respectively. The S1 subunit contains two subdomains, the N-terminal domain (NTD) and the C-terminal domain (CTD). In different coronaviruses, fragments of one or both subdomains can form the receptor binding domain (RBD). Analysis of the molecular structure of the protein shows the N- and C-terminus of S1 fold as two independent domains. According to currently available high-resolution crystal structure information, the RBD opens up, exposing a number of amino acid residues (shown in blue in Figure 1B) that directly contact the receptor amino acids [13,14,15].

Coronaviruses of different types use a wide range of receptors to enter target cells. Despite the highly conserved amino acid sequences in S1, various coronaviruses penetrate the cell by binding to S-proteins with different receptors. Thus, epidemiologically dangerous human HCoV coronaviruses interact with N-aminopeptidase (CD13) or N-acetyl-9-O-acetylneuraminic acid located on host epithelial cells. MERS-CoV penetrates the cell by interacting with DPP-IV dipeptidyl peptidase; SARS-CoV and SARS-CoV-2 bind to angiotensin-converting enzyme 2 (ACE-2) [2].

The SARS-CoV-2 spike protein binds its RBD to ACE-2 with high affinity and specificity. This could indicate that interfering with the RBD-ACE-2 interface can reduce the risk of infection [16]. The crystal structure of the RBD-ACE-2 complex has been previously described in some detail [17,18]. The RBD loop contacts the arc-shaped helix of the ACE-2 enzyme proteolytic domain. The domain–enzyme binding interface is divided into three contact zones in which amino acid residues on both sides form different intermolecular interactions (Figure 1C). The first contact zone is located on the N-terminus side, where amino acids Gln498, Thr500, and Asn501 form hydrogen bridges (shown with dotted yellow lines in the figure) with amino acid residues of the enzyme: Tyr41, Gln42, Lys353, and Arg357. The central part 1 of the enzyme helix and domain loops are contacted by the formation of a salt bridge between Lys417 RBD and Lys31 ACE-2 and a pi-stacking contact between aromatic rings of Tyr453 RBD and His34 ACE-2. At the C-terminus, Gln474 RBD contacts Gln24 of ACE2, and Phe486 RBD interacts with Met82 of ACE2 via intense hydrophobic and coulomb interactions [16].

Theoretically, the interaction between ACE-2 and RBD can be blocked by binding the above-described functional residues of the receptor and/or domain by potentially biologically active molecule, and thereby preventing viral entry. This is a good strategy. A previous review [16] cites work describing synthetic peptides, monoclonal antibodies, and chimeric proteins that are characterized by high affinity to the receptor-binding domain of the spike protein. For a number of reasons, peptide inhibitors have a number of drawbacks that limit their use as drugs, in particular, poor metabolic stability, poor membrane permeability, and rapid clearance [20,21]. Given this, the search for low-molecular-weight drugs that can either prevent the interaction of RBD with ACE-2 or affect the stability of the already formed complex is a very urgent task. We will not mention the low-molecular-weight compounds that have the ability to directly inhibit ACE-2 here; due to the importance of this enzyme in normal physiological processes, its direct inhibition cannot be considered as a successful therapeutic approach.

Arbidol (the same—Umifenovir) is an antiviral drug widely used in the Russian Federation for influenza therapy and is also licensed for use in China. According to [22], Arbidol demonstrates broad activity against several strains of influenza virus. In addition, according to [23], Arbidol effectively inhibits the SARS-CoV-2 virus in in vitro tests. The 50% maximum effective concentration (IC50) and 50% cytotoxicity (CC50) values for Arbidol were 4.1 µM and 31.8 µM, respectively. Another article [24] gave values of IC_50_ = 10 µM and CC_50_ = 20–100 µM. Arbidol shows maximum activity exactly in the first stages of infection, i.e., it can be considered as a virus entry inhibitor [24]. The antiviral effect of Arbidol against HCoV-229E and HCoV-OC43 seasonal coronaviruses in Vero E6 cells was demonstrated in a recently published paper [25].

**Figure 2:**
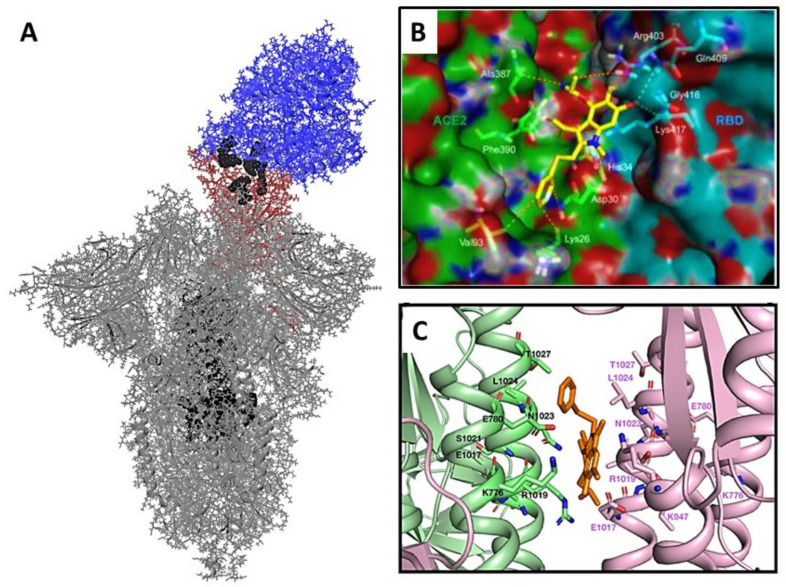
Potential binding sites based on previously published work: (**A**) general view of the S-protein of SARS-CoV-2 coronavirus (PDB code 7DF4 [28]) in an open conformation associated with ACE-2: amino acids belonging to the binding domain are shown in red, those belonging to the ACE-2 enzyme are shown in blue; amino acids belonging to the central part of the protein are shown in black; amino acids presented as a Van der Waals model can presumably participate in binding to the Arbidol molecule; (**B**) inset from the original article [26] showing the location of Arbidol in the domain-enzyme binding interface; Reprinted from ref. [26], Copyright 2021 Elsevier (**C**) inset from the original article [27] showing the putative site of Arbidol interaction with amino acid residues in the central protein part; Reprinted from ref. [27], Copyright 2021 Elsevier.

The interface between RBD and ACE-2 is considered a potential binding site for Arbidol [26] (Figure 2A,B). The authors suggest that the molecule forms a series of strong interactions with amino acid residues of the domain and enzyme, stabilizing the domain–enzyme complex, which leads to a decrease in the structural flexibility of the protein and causes difficulties in penetration into the host cell. According to theoretical research residues Lys 26, Asp 30, His 34, Val 93, Ala 387, Pro 389, and Phe 390 contributed the most to the ACE2 affinity for arbidol. According to theoretical research [27], residues Lys 26, Asp 30, His 34, Val 93, Ala 387, Pro 389, and Phe 390 contributed the most to the ACE2 affinity for arbidol. On another side, Arbidol exhibits a high affinity for RBD due to interaction with amino acids, such as Arg403, Asp405, Glu406, Gln409, Gly416, Lys417, Ile418, and Tyr505. Some of these amino acids are located in the central contact area ACE-2 and RBD (Figure 1C). On the other hand, it is known [28] that Arbidol binds to the stem portion of influenza virus haemagglutinin, preventing the transition from pre- to post-fusion conformation and, consequently, the fusion of the viral and cell membrane. Influenza haemagglutinin and the S-spike protein of the coronavirus SARS-CoV-2 belong to type I surface proteins with a similar fusion mechanism. The presence of similar heptad repeats in these proteins suggests similar hydrophobic cavities in the space between the α-spirals of the stem part of the protein. Based on the analysis of the HA2 and S2 subunit protein sequences, the author [27] identified a potential binding site for Arbidol in a small region of the S2 domain of the coronavirus. Arbidol is located between the trimerization helices of two protomers of S-protein: K776, R1019, N1023, L1024, T1027 (Chain A), and E780, K947, E1017, S1021, L1024 (Chain B). It is assumed that Arbidol interacts with these key amino acid residues of the stem part and effectively blocks or prevents S-protein trimerisation (Figure 2A,C), which plays an important role in the host cell entry process. On the other hand, the authors of two publications used only molecular modelling techniques without any experimental confirmation.

Analysis of the above sources raises additional questions: Is their evidence that Arbidol exhibits activity at the stage SARS-CoV-2 virus entry stage into the host cell and, if so, where exactly is it indicated that the antiviral agent can bind to the virus glycoprotein?

In recent years, along with conventional, replication-competent viruses, pseudoviral systems have been actively used to detect agents that specifically inhibit certain viral proteins [29]. A pseudovirus is a recombinant particle consisting of the capsid of one virus (usually lentivirus or vesicular stomatitis virus) with proteins of another virus on its surface. Binding and penetration of such a pseudoviral particle into the cell is fully provided by the surface protein. Pseudotyping is achieved using plasmids encoding various surface proteins. The final step in the production of pseudoviruses, namely, the budding of the pseudoviral particle from the surface of the producer cell, is similar to that of conventional viruses, and the protein responsible for this step plays a key role. The advantages of the pseudoviral system are the ability to identify the stage of the virus life cycle at which inhibition occurs and its high biological safety.

In the presented work, we tested the efficacy of Arbidol using the pseudoviral system we have developed; the system we used is based on the standard lentiviral pseudotyping system. This allows for obtaining viral particles pseudotyped by the SARS-CoV-2 surface protein. Such particles mimic the penetration system of the natural virus but are not capable of replication, i.e., they are single-cycle viruses. Compared to traditional assays, pseudovirus assays have shown good correlation with live virus assays, and are generally more productive and take less time to experiment [30,31]. In addition, we would like to explain the mechanism of the antiviral action of Arbidol using modern molecular modelling techniques. In this case, we have significantly changed the “template” approach to theoretical research. In most cases, authors often use molecular docking methods in combination with molecular dynamics methods exactly in this sequence. This approach certainly works if the authors are confident about the binding site of the active ligand in the potential biological target. In the case of the description of the mechanism of the antiviral action of Arbidol, the binding site of the latter is not reliably determined. In this work, using molecular dynamics methods, we will try to create a model of interaction of several molecules of Arbidol with receptor-binding domain and RBD-ACE-2 complex somewhat closer to the real experiment. Moreover, we considered interaction Arbidol with the glycosylated system at position Ans343. Of course, our approach is not new in principle; thus, we use the method of multi-ligand implementation [32] to identify and characterize potential binding sites for 10 different biological targets. In another paper [33], the authors, using combined molecular dynamics methods, identify the most likely ligand and biological target binding sites. However, similar approaches to explain the mechanism responsible for the antiviral activity of biologically active compounds have not been previously found in the literature.

Our research team is currently making significant efforts in the search for effective inhibitors of SARS-CoV-2 virus entry using terpene series compounds as initial building blocks [34]. Our work has shown that the above strategy can be quite successful [35,36,37,38]. The aim of the present study is to validate the pseudoviral system we have developed, identify the activity of Arbidol against the pseudoviral system, and statistically determine the potential binding site of Arbidol. It is also important to find out whether the binding of Arbidol at alternative sites of the receptor-binding domain and/or the domain–enzyme complex affects the conformational structure of the latter. Moreover, a probable binding site located in the S1 subunit in the region of the fusion peptide (FP2) was also considered. Modern clinical trials have shown [39,40] that umifenovir monotherapy appears to be ineffective. However, we are considering Arbidol as a potential comparison drug because no other antiviral agents active in the early stages of viral replication are currently available. Moreover, crystal structures of complex RBD S-protein with potential ligands are currently absent, which limits the development of effective antiviral compounds now at this moment. We hope that the potential binding sites found may be suitable and useful for following design for new efficient inhibitors of virus entry.

## 2. Materials and Methods

### 2.1. Biological Experiments

#### 2.1.1. Cell Cultures

The HEK293T cell line was provided by the Department “Collections of Microorganisms” of the Rospotrebnadzor State Research Center Vector (Koltsovo, Russia). Cells were cultured on Dulbecco Modified Eagle Medium (DMEM) (Invitrogen, Carlsbad, CA, USA) and Dulbecco Modified Eagle Medium/Nutrient Mixture F-12 (DMEM/F12) (SRC Vector, Koltsovo, Russia), with the addition of 10% (*v*/*v*) thermally inactivated foetal cow serum (Invitrogen, Carlsbad, CA, USA), and 0.6 mg/mL L-glutamine (Invitrogen, Carlsbad, CA, USA) and 50 µg/mL gentamicin.

#### 2.1.2. Plasmids

A second-generation lentiviral system was used to generate pseudoviruses. psPAX2, which provides formation of lentiviral particles (Addgene #12260), was used as a packaging plasmid. The Ph-SΔ18 encoding the SARS-CoV-2 protein was used as the envelope plasmid and was obtained by inserting the nucleotide sequence encoding the S protein SARS-CoV-2 (GenBank:MN908947) into the phMGFP vector. The last 18 amino acids of the S protein sequence were deleted, and then the codon composition was optimized using the GeneOptimizer tool (https://www.thermofisher.com/ru/en/home/life-science/cloning/gene-synthesis/geneart-gene-synthesis/geneoptimizer.html, accessed on 20 February 2020). The final nucleotide sequence was synthesized by DNA-Synthesis LLC. The insertion was performed at the NheI and AsiGI sites. The reporter plasmid pLenti-Luc-GFP was obtained from the lentiviral vector pCDH-EF1a-GaussiaSP-MCS-IRES-copGFP (kindly provided by T.N. Belovezhets, (ICBFM SB RAS) by replacing the Gaussia luciferase sequence with that of firefly luciferase. For this purpose, PCR amplification of the firefly luciferase nucleotide sequence was performed using the primers Lenti-Luc-F 5′-aaaaaatctagctagccaccatggaagatgcca-3′ and Lenti-Luc-R 5′-aaaaaaggatccttacacggcgatcttgccg-3′. Plasmid pCAG-luciferase (Addgene #55764) was used as a matrix. Next, the PCR product was inserted into the pCDH-EF1a-GaussiaSP-MCS-IRES-copGFP plasmid at the XbaI and BamHI restriction sites. To obtain a culture of cells HEK293T transiently expressing ACE2, we used the pCAG-ACE2 plasmid. To obtain plasmid pCAG-ACE2, PCR amplification of the ACE2 nucleotide sequence was performed using primers HeC-F 5′-aaaaaaGCTAGCccaccatgggatggtcatgtat-3′ and HeC-R 5′-cagaggttgattgtcgactaaaagg-3′. Next, the PCR product was inserted into the pCAG-Luc plasmid at the AsuNHI and SalI sites.

#### 2.1.3. Preparation of SARS-CoV-2 Pseudotyped Lentiviral Particles

To obtain SARS-CoV-2 pseudotyped lentiviral particles, we co-transfected HEK293T cells in T75 matrices with three psPAX2 (10 µg), ph-SΔ18 (10 µg), and pLeni-Luc-GFP (10 µg) plasmids in a 1:1:1 ratio. Lipofectamine 3000 (2 μL per μg of plasmid) (Invitrogen, Carlsbad, CA, USA) was used as a transfectant. The transfected HEK293T cells were incubated at 37 °C in an atmosphere of 5% CO_2_ for 2 days, after which the supernatant containing lentivirus particles coated with SARS-CoV-2 protein were collected and filtered through a 0.45 μm filter (Millipore, Burlington,MA, USA). After filtration, 500 µL aliquots were made and stored at −80 °C.

#### 2.1.4. Determination of the Ability of SARS-CoV-2 Pseudotyped Lentiviral Particles to Penetrate HEK293T Cells

Fifty μL of trypsinised suspension of HEK293T cells after transfection with pCAG-ACE2 plasmid at a concentration of 1 × 10^6^ cells/mL medium was added to a 96-well plate. Then, 50 μL of pseudovirus-containing supernatant was added to the cells in four replicates. After 48 h, the luminescence level was determined using the Luciferase Assay System (Promega, Madison, WI, USA). After the growth medium was removed from the pseudovirus-infected cells, they were lysed using 1× cell culture lysis buffer (50 μL/well) (Promega, Madison, WI, USA). Then, 35 μL of lysate was transferred into black optical plates, and the luminescence level was measured on a Varioskan LUX instrument (Thermo Fisher Scientific Inc., Waltham, MA, USA) with the automatic addition of luciferase substrate (35 μL/well).

#### 2.1.5. Determination of Cytotoxicity of Compounds on HEK293T Cells

To determine the cytotoxic concentration of compounds, the day before compounds were added to 96-well culture plates, HEK293T cells were seeded in an amount of 100 μL cell suspension per well (10^4^ cells per well) and placed in a CO_2_ incubator. The next day, after 24 h of incubation, different concentrations of test compounds were added to the cell culture by sprouting (initial concentration of 1 mg/mL). Each concentration was tested in three replicates. DMSO at a concentration of no more than 1% was added to the control wells. The final volume of medium in the well was 200 µL. The plate with added compounds was incubated in a CO_2_ incubator for 72 h at 37 °C and 5% CO_2_. After 72 h of incubation of the cell line with the tested compounds, 20 µL of MTT working solution (5 mg/mL) was added to each well and incubated for another 2 h under CO_2_ incubator conditions. After 2 h, plates were removed from the CO_2_ incubator, and the medium in each well was replaced with DMSO solution (50 µL/well). The plates were gently shaken to dissolve the formazan crystals. The optical density of each well at 570 nm was determined using a plate reader. The survival of HEK293T cells in the presence of the test substance was calculated using the formula: (OD of experimental wells − OD of medium)/(OD of control wells − OD of medium) × 100%, where OD is the optical density. The concentration causing 50% cell death (CC_50_) was determined from dose-dependent curves using GraphPad Prism 6 software. A range of nontoxic concentrations was chosen for each compound in which antiviral activity was investigated.

#### 2.1.6. Determination of Semi-Inhibitory Concentrations of Compounds against Lenti-S SARS-CoV-2 Pseudoviruses and Calculation of Selectivity Index (SI) Values

To determine the inhibitory capacity of the compounds tested, a neutralization assay was performed using HEK293T cells and lentiviral particles exhibiting S protein of SARS-CoV-2. Briefly, serial dilutions of the compounds in DMEM culture medium (without serum or antibiotic) were prepared in 96-well plates. Then, suspension of pseudoviruses (50 μL/well) was added to the diluted compounds, and the mixture of compounds with pseudoviruses was incubated in a CO_2_ incubator for 1 h at 37 °C and 5% CO_2_. After 1 h, HEK293T cells (pCAG-ACE2 transfected) (1.5 × 10^4^ cells/well) were added and incubated at 37 °C in 48 h. The assay was performed in three replicates. The infectivity of pseudoviruses in the presence of inhibitors and in control (uninhibited) samples was determined by the luminescence index 48 h after infection. The percentage of neutralisation of each sample was calculated as the ratio between the RLU values of the test wells (test sample + pseudovirus + cells) and the virus control (pseudovirus + cells). Statistical data processing and IC_50_ calculations were performed using GraphPad Prism 6 software using the nonlinear regression method. After the IC_50_ was determined, the Selectivity index (SI)—the ratio of compound toxicity and inhibitory activity against the virus (CC_50_/IC_50_)—was calculated.

### 2.2. Molecular Dynamics and Docking

#### 2.2.1. Ligand and Protein Preparation

The geometric parameters of the RBD-ACE-2 complex and a full-size spike-protein were downloaded from the protein data bank database [19], PDB ID code 6VW1 [2], and 7BNM [41]. The PBD ID 6VW1 contains information about glycosylated protein. Only RBD glycan at position Asn343 was considered. Carbohydrate residue was adjusted according to [42]. To simulate the interaction process event, the RBD structure and the complex including RBD-ACE-2 were studied separately. All protein models were prepared before calculations: missing hydrogen atoms were added, and crystallization water was removed. Restrained minimization was processed in order to optimize the network of hydrogen bonds in the structure of observed proteins [43]. The geometric parameters of the proteins and small molecules (Arbidol) were calculated with use of the OPLS3e/OPLS4 force field [44]. All manipulations and following calculations were performed in a Schrodinger Suite 2021-2 program package.

#### 2.2.2. Molecular Dynamic Models

The process of interaction of Arbidol (Arb) with RBD protein and ACE-2-RBD protein-protein complex was simulated as follows: the systems RBD-nArb and ACE-2-RBD-nArb, where *n* = 1, 5, 10, 15, and 20 (number of Arbidol molecules) were arranged in an orthorombic boundary box, filled with 0.15 M aqueous NaCl solution (isotonic saline). Arbidol molecules were placed at some random distance from the protein, excluding any contact with the protein surface (Figures S1–S6). RBD-SUG-n×Arb (*n* = 1, 20) was considered glycosylated systems. In this case, three full-size S-protein Arbidol molecules were placed in the region of heptad repeats of the subunit S2 of S-protein. This place was chosen according to theoretical research by Vankadari [27]. The buffer zone size was 15 Å from the protein surface for all observed molecular models. In addition, we examined the behavior of 15 Arbidol molecules in a physiological solution without protein. In all simulations, as the solvent used a TIP3P water model. The environment was NPT (constant pressure, temperature, number of particles). The period of recorded simulated dynamics was 300 nanoseconds at temperature 310 K (37 °C). The protocol of system preparation for simulation includes pre-minimization and equilibration stages: simulate in the NVT ensemble with Brownian dynamics at 10 K with small time steps and solute non-hydrogen atoms restrained; simulate in the NVT ensemble using a Langevin thermostat with: simulation time of 12 ps, temperature of 10 K fast temperature relaxation constant velocity resampling every 1 ps, non-hydrogen solute atoms restrained; simulate in the NPT ensemble using a Langevin thermostat and a Langevin barostat with: simulation time of 12 ps, temperature of 10K and a pressure of 1 atm, fast temperature relaxation constant, slow pressure relaxation constant velocity resampling every 1 ps, non-hydrogen solute atoms restrained; simulate in the NPT ensemble using a Langevin thermostat and a Langevin barostat with: simulation time of 12 ps, temperature of 300 K and a pressure of 1 atm, fast temperature relaxation constant slow pressure relaxation constant, velocity resampling every 1 ps, non-hydrogen solute atoms restrained; simulate in the NPT ensemble using a Langevin thermostat and a Langevin barostat with: simulation time of 24 ps, temperature of 300 K and a pressure of 1 atm, fast temperature relaxation constant, normal pressure relaxation constant. After that, the main simulation was started, with recording of 10,000 frames for each MD simulation. All calculations were performed with Desmond module, included in Schrodinger suite 2021-2.

For the RBD-n×Arb and ACE-2-RBD-n×Arb systems (*n* = 1, 20), we performed additional simulations of 100 ns each at 400 K. The end state of the previous simulations was used as the starting point of the following simulations. Increasing the temperature allows us to cover a larger field of conformation states of the system in a shorter computation time interval.

#### 2.2.3. Population Analysis

Population analysis was performed using the VolMap plug-in implemented in the VMD program [45]. Two types of maps based on weighted atomic density and weighted atomic population were considered to determine the statistical binding site of Arbidol to the proteins in question. The smaller the cloud size, the lower the probability of finding the molecule in that region of space. At the same time, attention was paid to several amino acid residues of the protein, whose interaction with Arbidol molecules is unlikely due to steric difficulties (Figure S7).

#### 2.2.4. Analysis of Secondary Structure Change

To quantify protein structure changes before and after simulation, the MultiSeq plugin implemented in the VMD program was used. The RMSD parameter was chosen as the change descriptor. The circuits (ACE and RBD) were analyzed separately. The changes were visually represented as a color gradient (Blue-White-Red). RMSD per residue plots were also formed for the chains (ACE and RBD) separately.

#### 2.2.5. Interaction Interface Analysis

The domain-receptor contact region is divided [16,17] into three zones, where a few amino acid residues on one side form different intermolecular interactions with the other (Figure 1C). In [26], the following amino acids were marked as making contact on the domain side: Arg403, Tyr449, Tyr453, Phe490, Tyr473, Ala475, and Gly476. Arbidol binds to the ACE-2 contact site predominantly with Arg403, Gln409, Gly416, and Lys417 on the RBD domain side and with Ala386, Phe390, Val93, Lys26, Glu30, and His34 on the enzyme side, forming a series of intermolecular contacts.

When analyzing the interaction of the Arbidol molecules with the surface of the domain and the domain–enzyme complex, we paid special attention to the contact zone between the domain and the enzyme, in the region of amino acids 437 to 508 (Figure 1C). The region close to fusion peptide 2 was considered more thoroughly in the case full-size protein.

#### 2.2.6. Molecular Docking

Population areas or contact sites of Arbidol molecules that are statistically more frequent were selected for the molecular docking procedure. Arbidol docking was performed using a forced ligand positioning protocol (Glide induced-fit docking or Glide IFD, implemented in Schrodinger Suite 2021-2 program [46]) with the following conditions: flexible protein and ligand, 15 Å grid matrix size, amino acids within 5 Å of the ligand were constrained to be optimized for ligand influence. Docking solutions were ranked by evaluating the following calculation parameters: docking score (based on GlideScore with penalty exclusion), ligand efficiency (LE, where the per-heavy-atom distribution of the scoring function is considered), and the model energy value parameter (Emodel), including GlideScore value, energy of unbound interactions and energy parameters spent on the formation of compound stacking in the binding site. Binding energies (ΔG_MM-GBSA_) of ligand–protein complexes were estimated using the variable-dielectric generalized Born model for best docking positions. The solvent was water (implicit).

## 3. Results and Discussion

### 3.1. Biological Experiments

**Figure 3:**
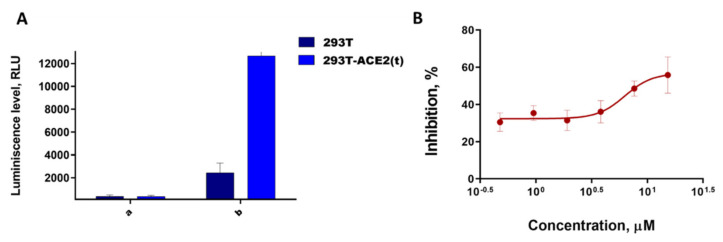
(**A**) SARS-CoV-2 lentiviral pseudovirus infectivity (a—background luminescence of cells without adding pseudoviruses; b—the level of luminescence of cells after the introduction of pseudoviruses); (**B**) dependence of SARS-CoV-2 pseudovirus penetration inhibition degree on Arbidol concentration in the medium.

To assess the inhibitory activity of Arbidol, we used lentiviral particles carrying the SARS-CoV-2 protein S on their surface. Since it is known that pseudoviruses containing SARS-CoV-2 S protein on their surface either do not infect the original HEK293T cells at all, or infected with low efficiency [47]; additionally, for analysis, we obtained HEK293T cells displaying angiotensin converting enzyme II (ACE2) on their surface by transient transfection-HEK293T-ACE2 (t). We then evaluated the SARS-CoV-2 pseudovirus for infectivity in HEK293T cells and HEK293T-ACE2 (t) cells. As expected, our SARS-CoV-2 pseudoviruses had practically no infectivity in HEK293T cells due to the absence of the ACE2 receptor, while HEK293T-ACE2 (t) had a signal 5 times higher than background (Figure 3A).

Using the obtained lentiviral particles carrying the SARS-CoV-2 virus glycoprotein on their surface, we analyzed the activity of Arbidol and another virus entry inhibitor, Maraviroc. Maraviroc is an HIV virus inhibitor that interacts with the CCR5 chemokine receptor and is active at the early stages of viral replication [48]. We have shown that, under the conditions of our experiment, Arbidol shows an activity at the IC_50_ dose of 8.32 ± 3.06 µM (Figure 3B) and has cytotoxicity on HEK293T cells at the CC_50_ dose of 30.99 ± 3.85 µM. Maraviroc used for HIV therapy shows no activity at all against the pseudoviral system. Our research independently confirms that Arbidol has activity against SARS-CoV-2 virus, but the selectivity index responsible for the efficacy of this agent is not high. Moreover, our IC_50_ value falls within the range of previously published values characterizing the effectiveness of Arbidol of 4.1 µM [23] and 10.0 µM [24].

Based on these biological experiments, we cannot describe the direct molecular mechanism of Arbidol antiviral action, but we can consider it as a possible entry inhibitor. We also cannot assume a potential binding site to the surface of the spike protein. However, using combinations of molecular modelling techniques, we can infer a likely binding site for Arbidol, for example, in the receptor-binding domain of the surface protein.

### 3.2. Molecular Dynamics

For all the systems studied, the change in RMSD values for protein structures (both domain and domain–enzyme complex) is not significant, but RMSD of all systems showed normal equilibration, which means acceptable quality of simulated systems. Fluctuations in RMSD values within 2–4 Å are considered acceptable for most globular proteins (Figures S8–S17). Attention should be paid to the stability of the positions of the Arbidol molecules in the multi-ligand system, which is explained by its tendency to form aggregates. According to [49], as an indole derivative, Arbidol is poorly soluble in water, which strongly affects its bioavailability and pharmacokinetics. The tendency of Arbidol to form agglomerates in physiological solutions observed in molecular dynamics simulations correlates with the data on its poor solubility and low bioavailability (Figure S18).

#### 3.2.1. Molecular Dynamic of RBD-n×Arb and RBD-ACE-2-n×Arb Unglycosylated Systems

In this work, the main goal of molecular dynamic simulations is to search for statistically significant binding sites of Arbidol to the surface of the domain and/or to the surface of the domain–enzyme complex. The analysis of occupancy maps based on weighted atomic density and weighted atomic occupancy allows for determining which amino acid sequences of proteins preferentially form intermolecular interactions with Arbidol. In addition, statistical processing of the geometric parameters of proteins allows us to estimate the degree of influence of the ligand on the secondary structure of the protein.

**Figure 4:**
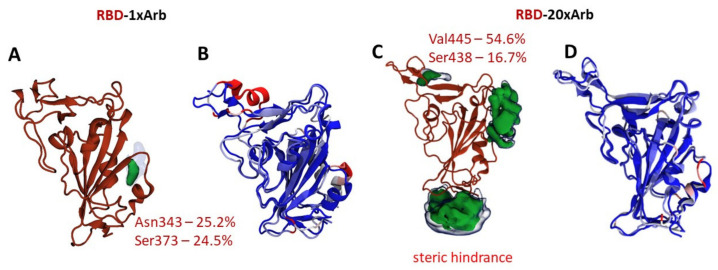
Statistical processing of molecular dynamics trajectories of systems, based on RBD-1×Arb and RBD-20×Arb: (**A**,**C**) occupancy maps, showing site location, percentile and area of protein–ligand interactions between RBD and Arbidol molecules during simulation time; (**B**,**D**) structure fluctuation in receptor-binding domain in the presence of Arbidol. Blue-to-Red gradient on secondary structure elements shows us intensity of structural changes in the presence of multiple arbidol ligands (Blue—no changes, Red—maximal change. Measured by RMSF value calculations).

**Figure 5:**
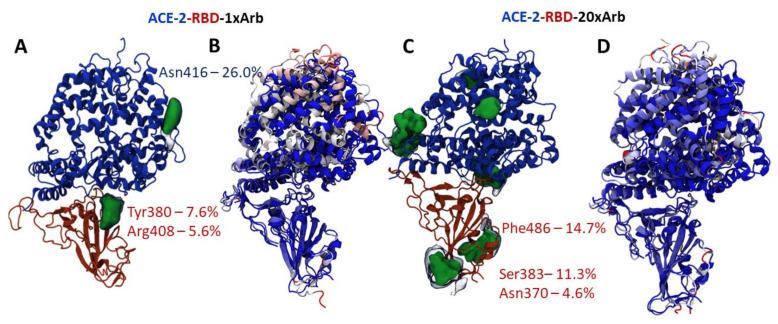
Statistical processing of molecular dynamics trajectories of systems, based on systems with ACE2-RBD-1×Arb and ACE2-RBD-20×Arb: (**A**,**C**) occupancy maps, showing site location, percentile and area of protein–ligand interactions between RBD/ACE-2 and Arbidol molecules during simulation time; (**B**,**D**) structure fluctuation in receptor-binding domain in the presence of Arbidol. Blue-to-Red gradient on secondary structure elements shows us the intensity of structural changes in the presence of multiple arbidol ligands (Blue—no changes, Red—maximal change. Measured by RMSF value calculations).

Figure 4 and Figure 5 are showing the occupancy maps (Figure 4A,C and Figure 5A,C), and analysis of the changes in the secondary structure of the proteins (domain and domain–enzyme complex) during the molecular dynamic simulation time (Figure 4B,D and Figure 5B,D). Information is given here for single-molecule Arbidol proteins and for the RBD-20×Arb and ACE-2-RBD-20×Arb multi-ligand systems. The size of the population “cloud” depends on the frequency of the ligand appearance near the indicated protein surface location as well as the ligand retention time at that position. The population maps for the remaining systems studied are presented in the accompanying material (Figures S19–S21 and Tables S1 and S2).

In the RBD-1×Arb system, Arbidol most often forms protein–ligand interactions with Asn343 and Ser373. These amino acids are not included in the contact region of the receptor-binding domain, but they are also not among the forbidden amino acids whose interactions are excluded due to structural constraints (because this fragment of protein and forbidden aminoacids is covered by another, not showed, domains/subunits). For the multi-ligand system, occupancy clouds appear more frequently at the interface of the domain–enzyme interaction, contacting Val445 and Ser438 (Figure 4C).

In the ACE-2-RBD-1×Arb system, Arbidol can interact with both the enzyme (Asn416) and the domain in the Tyr380 and Arg408 regions (Figure 5A). In this case, the probability of finding an Arbidol molecule near the surface of the enzyme is slightly higher than that near the surface of the domain. In the multi-ligand system, the probabilities of the formation of intermolecular interactions between the Arbidol molecules and the amino acids of the enzyme and the domain are approximately equal.

Analysis of changes in the secondary protein structure is another opportunity to assess the site of potential interaction of Arbidol with proteins. In systems with a single molecule of Arbidol, changes in the secondary structure are more dramatic. In the case of the enzyme-domain complex, it is the enzyme that is most affected by Arbidol (Figure 4B and Figure 5B show the most significant changes in red). In multi-ligand systems, the effect of ligands on proteins is less pronounced (Figure 4D and Figure 5D).

#### 3.2.2. Molecular Dynamics of RBD-n×Arb Glycosylated Systems

Based on theoretical studies, we note that Arbidol (or other potential inhibitors of S-spike with similar pharmacophore features) can bind in the domain region close to Asn343. According to [14,50,51], this acid is one of RBD glycosylation sites; therefore, we carried out an additional molecular dynamics simulation of RBD-SUG-1×Arb and RBD-SUG-20×Arb systems for 300 ns (starting systems are given in SM, Figure S6, RMSD—Figures S16 and S17).

**Figure 6:**
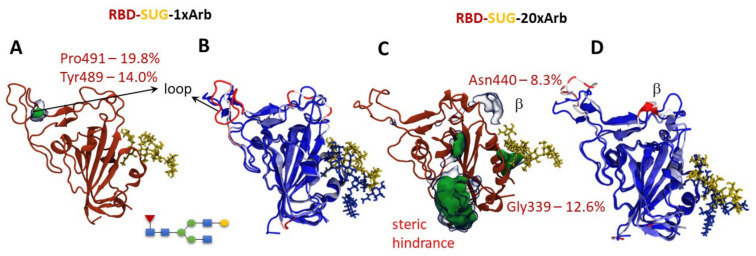
Results of molecular modeling of glycosylated systems RBD-SUG-1×Arb and RBD-SUG-20×Arb: statistical processing of the molecular dynamics simulations: (**A**,**C**) occupancy maps, showing site location, percentile, and area of protein–ligand interactions between RBD and Arbidol molecules during simulation time; (**B**,**D**) structure fluctuation in receptor-binding domain in the presence of Arbidol. Blue-to-Red gradient on secondary structure elements shows us intensity of structural changes in the presence of multiple Arbidol ligands (Blue—no changes, Red—maximal change, measured by RMSF value calculations).

The occupation map of RBD-SUG-1xArb shows that Arbidol contacts the protein surface in the loop area to form significant intermolecular interactions with the amino acid residues of Pro491 and Tyr489 (Figure 6A). This loop (highlighted in red in Figure 6B) is influenced by the ligand to a noticeable extent. On the one hand, a simulation of the UN-glycosylated system RBD-1×Arb (over 300 ns) shows that the ligand is more likely to contact the protein in the Asn343 region (Figure 4A). It should be noted here that the molecular-dynamic simulation shows only the probability of contact of Arbidol with the surface of the protein. Therefore, these observations should be interpreted according to the totality of the data obtained. This means that, when a single Arbidol molecule interacts with the RBD surface of UN-glycosylated and glycosylated systems, the domain loop (Arg454; Thr470; Tyr489; Pro491, see Figure 6A,B) and the cavity near the glycosylation site (Asn343; Ser371; Asn370) should be considered as the most probable binding sites.

An increase in of the number of Arbidol molecules in the system leads to a change in the contact zones of the ligands and the protein due to the tendency of Arbidol to form agglomerates (Figure S18). By data set, we can note amino acids located in the RBD-ACE-2 interface close to N-terminate (Val445, Se438, and Asn388) and in the glycosylation site (Gly339) (Figure 6C) despite the presence of a carbohydrate residue at position Asn343. In this case, the Arbidol effect on the domain loop was somewhat reduced. A greater change in the secondary structure is observed in the β-sheet in the Asn440 region (Figure 6D). In other words, areas of protein are available for contact with Arbidol despite the presence of carbohydrate residue. These are very logical results because carbohydrate residue is a very mobile moiety molecule. Moreover, Arbidol is rich in various pharmacophore groups such as aromatic rings, donor–acceptor interaction areas.

#### 3.2.3. Molecular Dynamics of Full-Size Proteins with Three Arbidol Molecules

**Figure 7:**
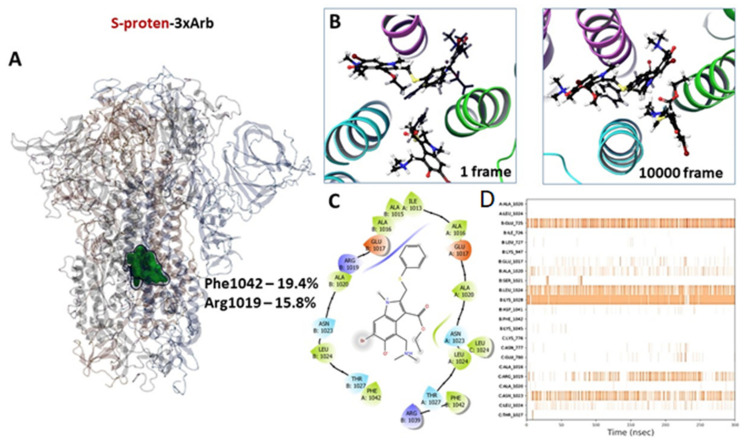
Results of molecular modeling of a complex of full-size protein and three molecules of Arbidol: (**A**) occupancy map, showing site location and protein–ligand interactions percentile in simulation; (**B**) initial and last in simulation, ligand positions in the stem domain of the subunit S2; (**C**) potential binding site, hydrophobic amino acids are presented by green sheets; polar residues are presented by blue; negative and positive charge amino acids are shown orange and violet, respectively; (**D**) the intensity of contacts between the ligand and amino acids during simulation time.

According to [27], Arbidol binds to the stem portion of the subunit S2 of a spike-protein analogous with the HA-Arbidol complex [28]. The binding site of molecules is located close to the fusion peptide place (Figure 7A). Three molecules bind with protein in such a way that each molecule contacts amino acids of two monomers.

Molecules do not leave the potential binding site within 300 ns of molecular dynamics simulation, but they shift from the start position (Figure 7B) to each other, forming an agglomerate of three molecules. The tendency to form Arbidol agglomerates has been discussed above. The potential binding site is saturated with hydrophobic amino acids such as alanine, isoleucine, leucine, and phenylalanine (Figure 7C). Here, similarities are observed with binding sites of inhibitors of surface viral proteins, such as influenza virus hemagglutinin and Ebola virus glycoprotein [35]. Most often, Arbidol molecules contact Phe1042, Arg1019 and Asn1023. Hydrogen bonds are formed with Glu725, Leu1024, Asn1023, and Arg1019 (Figure 7D, Table S3).

### 3.3. Molecular Docking

Statistical processing of the molecular dynamics results allows us to identify potential binding sites for Arbidol on the protein surface. In the RBD-nArb and ACE-2-RBD-nArb, we identify several amino acid residues (Figure 4, Figure 5 and Figure 6) that can form intermolecular interactions with Arbidol molecules with a high probability (more than 5%, see Tables S1 and S2). Given the weighted atomic occupancy data, we identify several likely binding regions for Arbidol molecules on the protein.

**Figure 8:**
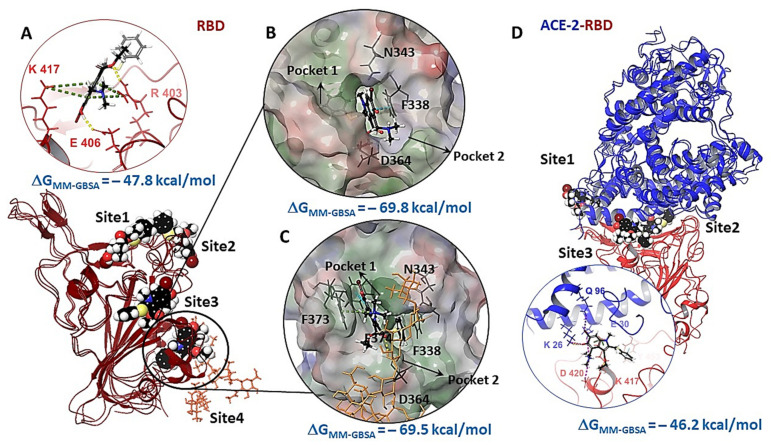
Molecular docking results to potential RBD binding sites: Hydrogen and salt bridges are shown with yellow and purple dashed lines, π-cation and π–π stacking interactions with green and blue dashed lines, respectively. (**A**) binding mode of Arbidol in the perhaps binding sites. Carbohydrate molecules are shown in orange; (**B**) Arbidol binding mode in pocket 1; (**C**) Arbidol molecule binding mode in pocket 2; (**D**) molecular docking results in potential binding sites of ACE-2-RBD complex.

In the case of RBD surface: one region is located at the interface of the domain–enzyme interaction and two are close to the glycosylation site near amino acid Asn343 (Figure 8A). In addition, we considered the possibility of Arbidol binding in the part of the protein described in [26]; this site refers to the contact site of the loops of the domain and the helix of the enzyme (Figure 1). According to the analysis of the results, the molecular docking affinity of Arbidol to the described binding sites is approximately the same, excluding site 4 (Figure 8A). Binding of Arbidol at this site is characterized by lower glide scores (additional energy parameters are presented in Tables S4 and S5, Figure S21).

Statistically, the Arbidol molecules tend to form intermolecular interactions with the amino acids Val445, Val446, and Gly496 (Figure 8A–site1) located in the β-loop domain and α-spiral enzyme contact region (Figure 1C). In terms of energy characteristics, the affinity of Arbidol is higher at the glycosylation site of the domain. At the same time, it should be noted that Arbidol molecules are virtually absent from the binding zone highlighted in green in Figure 8A and described in [26].

Site 4 is located close to Ans343. There are small hydrophobic pockets (Figure 8B,C) that can be attractive for binding ligands. In the UN-glycosylated systems case, the aromatic ring of Arbidol is in hydrophobic pocket 1, which is saturated with amino acids such as Val511, Leu513, Phe342, Phe338, Cys338, Leu355, etc. (Figure 8B), indole fragment forms π–π stacking with Phe338, while the carbonyl oxygen of the ligand binds with Asp364 by H-bridge. Protonated dimethylammonium forms hydrophobic contact with Val362, Leu224, Cys336, and Pro337 in the second pocket. If carbohydrate residues are present, non-significant steric hindrance occurs for the ligand. The molecule turns to locate the aromatic substituent of Arbidol in hydrophobic pocket 2 (Figure 8C). Perhaps, the hydrogen bridge between the carbonyl oxygen and NAG prevents Arbidol from sinking deeper into the hydrophobic pocket 1. Moreover, indole fragment forms π-π stacking with Phe374, and the protonated nitrogen forms pi-cation stacking with Phe373. In this case, the affinity of the ligand to the described binding site depends weakly on the presence of a carbohydrate residue (Table S4). In other words, Arbidol molecules can bind both in part of the domain–enzyme contact zone and at alternative sites on the protein surface.

In the domain-enzyme complex, the Arbidol molecule contacts the surface of the enzyme and the domain with approximately equal probability (Figure 8D and Figure S22). We selected two regions at the domain–enzyme contact site based on the statistics. The first site is located at the contact site of the domain loop and the enzyme helix, which includes the amino acids Phe486, and Ser477 on the domain site, and Thr82, Thr21, Gln87, and Tyr83 on the enzyme side. The second site chosen based on the simulation is located next to amino acid Lys61 of the enzyme, on the outer surface side of the protein-protein complex. The third site, located in the contact zone of the α-helix enzyme and domain loops (Figure 1), was chosen based on [26]. It should be noted here that simulations with different numbers of Arbidol molecules resulted in none of them falling within the region described in this article. Docking to potential cellular enzyme binding sites was not performed because the search for ACE-2 inhibitors is not the main goal of this work.

The affinity of Arbidol to the above sites is comparable. Therefore, based on the molecular docking data, it is difficult to say where the preferred binding site of Arbidol is. Considering the results of molecular dynamics simulations and the molecular docking procedure, we can see that Arbidol can bind both at the glycosylation site and at the domain-enzyme contact zone. This variation in binding sites cannot be regarded as contradictory data. Although the binding energy (ΔG_MM-GBSA_) of Arbidol at the site of glycosylation is more than 20 kcal/mol lower, obviously, the real situation of the interaction of Arbidol with the surface protein RBD is more complicated.

**Figure 9:**
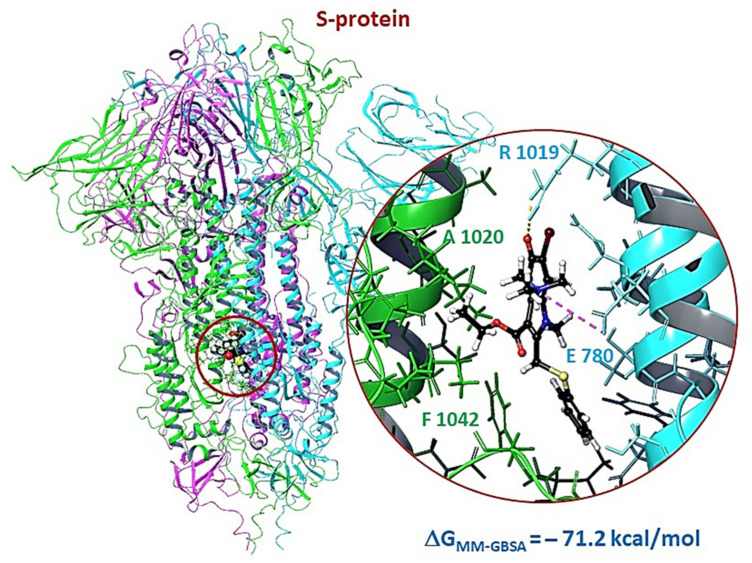
Results of molecular docking procedure of Arbidol in the binding site of the stem portion of the subunit S2: H-bond and salt-bridge are shown by yellow and violet dotted lines, respectively.

The energetic parameters of the affinity of Arbidol for the binding site in the stem part of subunit S2 are comparable with the values for the binding sites of RBD (Figure 9, Table S6). The molecule forms several intermolecular interactions: H-bond with Arg1019, salt-bridge with Glu780, and several hydrophobic contacts with Leu727, Val1040, Phe1042, Leu1024. The binding energy is −71.2 kcal/mol, which is comparable to the binding energies of Arbidol at the binding sites located near the glycosylation site.

To search for potential inhibitors of the receptor-binding domain of the spike protein, the RBD and ACE-2 binding interface is most often considered, which is reasonable. However, the question arises: can the active molecule bind elsewhere in the domain, affecting the conformation, the secondary structure of the protein, and thereby preventing or weakening the binding of the domain to the enzyme? Is it possible that more than one molecule plays a role in the process of domain inhibition? Why do most studies default to a 1-to-1 interaction? This can be understood if the potential biological target has a cavity accessible to only one molecule due to a few steric hindrances, as is the case for most enzymes. In the case of RBD, the situation is slightly different: we are investigating a surface viral protein. The receptor-binding domain is its very mobile part, which is activated to bind to the receptor in a certain conformation. Then, the question of where a potentially active molecule can bind remains open.

As a result of the molecular dynamic simulations performed in conjunction with molecular docking data, we can note the following fact: wherever a molecule or molecules of Arbidol binds, the interaction of the latter affects the structural flexibility of the protein. However, this contact can result either in a change in the shape of the interface when the domain and enzyme bind, or simply in a change in the conformational lability of the domain, which can subsequently affect its affinity to the enzyme. This could explain the antiviral activity of Arbidol.

In addition, the binding of Arbidol within the stem part of the domain cannot be ruled out. However, due to the large size of the molecular model, it is very difficult to apply the approach implemented to find binding sites in a domain or in a combination with it. The choice of a potential binding site for Arbidol can be made only on the basis that hemagglutinin of the influenza virus and glycoprotein of the coronavirus are in the surface viral proteins of type I. At the same time, given the low bioavailability of Arbidol and its tendency to form agglomerates, it is rather difficult to imagine that molecules can enter into the surface protein. The crystal structure of the HA-complex was obtained by treating HA with high concentrations of Arbidol [28]. Although this does not exclude the possibility of molecule binding at the stage of viral protein assembly in the ER, all of the above reasoning could explain the antiviral activity of Arbidol.

Thus, Arbidol can bind both to RBD itself and to the RBD-ACE-2 complex. In the first case, the contact of Arbidol with the surface of the domain may prevent its subsequent binding to the enzyme; in the second case, the consequence may be a weakening of the RBD-ACE-2 binding or, conversely, its strengthening. In any case, the above-described contacts of Arbidol with the surface domain may affect the functioning of the protein structure.

Arbidol can bind to the stem part of the domain and inhibit the fusion mechanism of the viral and cell membranes. The binding energies are commensurate for the site located near the amino acids Asn343 of RBD and near the site of the fusion peptide (FP2). There is nothing strange about this: these sites are saturated with hydrophobic amino acids. Therefore, the affinity of Arbidol to these places is higher.

Analysis of the scientific literature [23,24,26,27,52] and the results of molecular modeling show that Arbidol exhibits antiviral activity against SARS-CoV-2 due to the effect on the surface protein. This is directly demonstrated by experimental [23,24,52] and theoretical works [26,27]. The place of binding of Arbidol has still remained unknown. Unfortunately, there is no experimental evidence that Arbidol binds in the RBD or in the contact zone of the RBD and ACE-2, but also there is not any data to disprove that. Arbidol can bind in the stem part of the S2 domain of S-protein SARS-CoV-2 because Arbidol inhibits HA of influenza by binding exactly in the stem part of the HA2 domain [28]. HA and S-spike proteins are type I surface proteins with similar heptad repeats [53]. The process of fusion of the viral and cell membranes occurs after similar conformational rearrangements take place in the subunits of HA and S-protein. The cavities located between the α-helix of HP of these proteins are hydrophobic. Binding of a potential entry inhibitor in these cavities might inhibit conformational rearrangements. According to these conclusions, Ref. [27] chose this cavity of binding Arbidol in the stem part of the S-protein. In addition, the proteolysis-coupled mass spectrometry approach [52] shows that the likely binding site of Arbidol is located in a cavity between the α-helices of two monomers (amino acids 1021, 1024, 1027). The binding site was described earlier in [27]. According to our results of molecular dynamics, Arbidol most often contacts with 1024, 1028, and 1023 residues (Figure 7C). These facts allow us to assume that Arbidol can bind in the stem part of the S-protein SARS-CoV-2. The ability of binding of Arbidol with RBD (or contact zone of RBD and ACE-2) and in the stem of S2 at the same time is not excluded. Compounds that can similarly inhibit a surface protein are described [54] in the example of HA. Perhaps the low antiviral activity of Arbidol may be explained by its tendency to form agglomerates and bind in different places of the protein at the same time.

## 4. Conclusions

In this work, we created a working pseudoviral system with glycoprotein S on its surface and showed that Arbidol has activity at the early stage of SARS-CoV-2 virus replication, but the therapeutic index responsible for the effectiveness of this agent is not high. The antiviral activity of Arbidol may be associated with the suppression of surface protein functions.

Arbidol can bind to the surface of the RBD and/or in the stem part of the S-protein. Unfortunately, there is no clearer evidence that Arbidol binds only at one of the possible binding sites (or at all at the same time). However, there is no disproof either. At the moment, the crystal structures of complex of S-protein with ligands in the RBD or/and subunit 2 are absent. We hope that our theoretical studies will help in the informed selection of binding sites for potential inhibitors of coronavirus entry.

## Data Availability

Not applicable.

## Supplementary Materials

The following are available online at https://www.mdpi.com/article/10.3390/v14010119/s1. Figure S1: RBD-1×Arb and ACE-2-RBD-1×Arb, Figure S2: RBD-5×Arb and ACE-2-RBD-5×Arb, Figure S3: RBD-10×Arb and ACE-2-RBD-10×Arb, Figure S4: RBD-15×Arb and ACE-2-RBD-15×Arb, Figure S5: RBD-20×Arb and ACE-2-RBD-20×Arb, Figure S6: RBD-20×Arb and ACE-2-RBD-20×Arb, Figure S7: Spike-protein SARS-CoV-2 open conformation: hard-to-reach amino acids for Arbidol are shown with balls model, Figure S8: RMSD of ACE-2-RBD-1×Arb molecular dynamic simulation, Figure S9: RMSD of ACE-2-RBD-5×Arb molecular dynamic simulation, Figure S10: RMSD of ACE-2-RBD-10×Arb molecular dynamic simulation, Figure S11: RMSD of ACE-2-RBD-15×Arb molecular dynamic simulation, Figure S12: RMSD of ACE-2-RBD-20×Arb molecular dynamic simulation, Figure S13: RMSD of ACE-2-RBD-1×Arb molecular dynamic simulation, Figure S14: RMSD of ACE-2-RBD-20×Arb molecular dynamic simulation, Figure S15: RMSD of S-protein 3×Arb molecular dynamic simulation, Figure S16: RMSD of RBD-SUR-1×Arb molecular dynamic simulation, Figure S17: RMSD of RBD-SUR-20×Arb molecular dynamic simulation, Table S1: Molecular dynamics statistics for systems RBD-n×Arb, *n* = 1, 20 and RBD-SUG-n×Arb, (*n* = 1, 20), Table S2: Molecular dynamics statistics for systems ACE-2-RBD-n×Arb, *n* = 1, 20, Table S3: Molecular dynamics statistics for systems Spike-protein-3×Arb, Table S4: Docking results into the sites of PBD, Table S5: Docking results into the interface of RBD-ACE-2 complex, Table S6: Docking results into the subunits2. In addition, movies of the simulation are attached to the manuscript.
