# Supplementary material for: Simulation of Molecular Dynamics of SARS-CoV-2 S-Protein in the Presence of Multiple Arbidol Molecules: Interactions and Binding Mode Insights
Source: Viruses. 2022 Jan 10;14(1):119. doi: 10.3390/v14010119 (PMC8781717; doi:10.3390/v14010119)
Supplement: Supplementary file 1 [file viruses-14-00119-s001.zip › viruses-1508510-supplementary.pdf]

Simulation of Molecular Dynamics of SARS-CoV-2 S-Protein in the Presence of Multiple Arbidol Molecules: Interactions and Binding Mode Insights

Sophia S. Borisevich [1,*], Edward M. Khamitov [1], Maxim A. Gureev [2,3], Olga I. Yarovaya [4,*], Nadezhda B. Rudometova [5], Anastasiya V. Zybkina [5], Ekaterina D. Mordvinova [4], Dmitriy N. Shcherbakov [5],
Rinat A. Maksyutov [5] and Nariman F. Salakhutdinov [4]

1 Ufa Federal Research Center, Laboratory of Chemical Physics Ufa Institute of Chemistry, RAS, Octyabrya pr., 71, 450054 Ufa, Russia; khamitovem@gmail.com
2 Research Center "Digital Biodesign and Personalized Healthcare", I.M. Sechenov First Moscow State
Medical University, 119991 Moscow, Russia; max_technik@mail.ru
3 Department of Computational Biology, Sirius University of Science and Technology, 354349 Sochi, Russia
4 Department of Medicinal Chemistry, N.N. Vorozhtsov Novosibirsk Institute of Organic Chemistry SB RAS, Lavrent'ev Av., 630090 Novosibirsk, Russia; mordvinova97@mail.ru (E.D.M.); anvar@nioch.nsc.ru (N.F.S.)
5 State Research Center of Virology and Biotechnology VECTOR, Rospotrebnadzor, Koltsovo, 630559 Novosibirsk, Russia; andreeva_nb@vector.nsc.ru (N.B.R.); zybkina_av@vector.nsc.ru (A.V.Z.); dnshcherbakov@gmail.com (D.N.S.); maksyutov_ra@vector.nsc.ru (R.A.M.)
* Correspondence: monrel@mail.ru (S.S.B.); ooo@nioch.nsc.ru (O.I.Y.)

Supplementary materials

Molecular dynamics

Initial systems used for calculations

Hereinafter, the Arbidol molecule is shown in the form of the van der Waals model; RBD is red; ACE-2 is blue; sodium cation and chloride anion are shown green big and dark-red small balls, respectively.

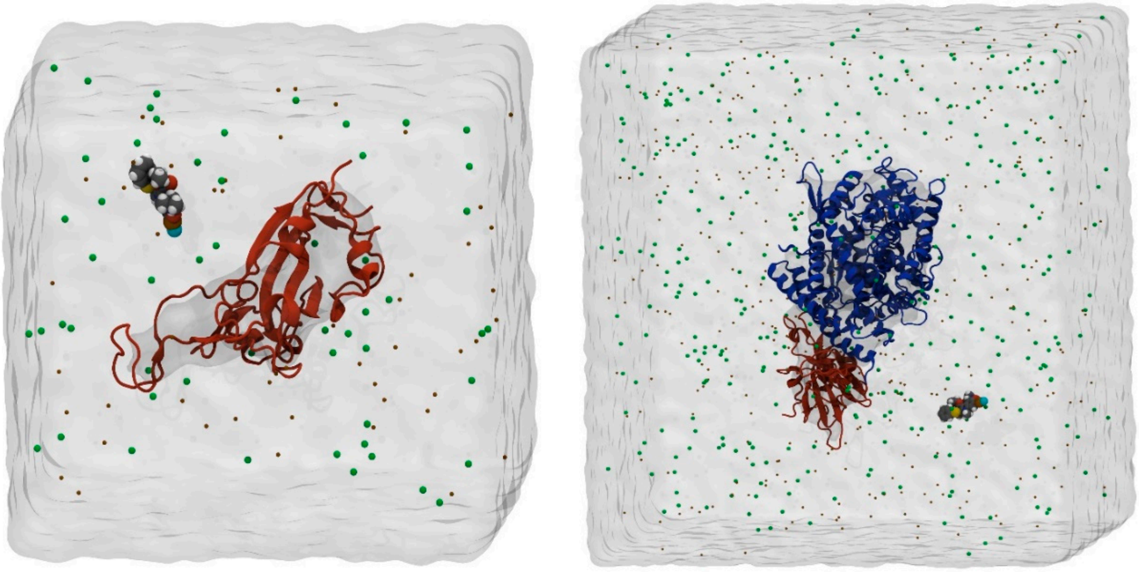

Figure-S1 RBD-1×Arb and ACE-2-RBD-1×Arb

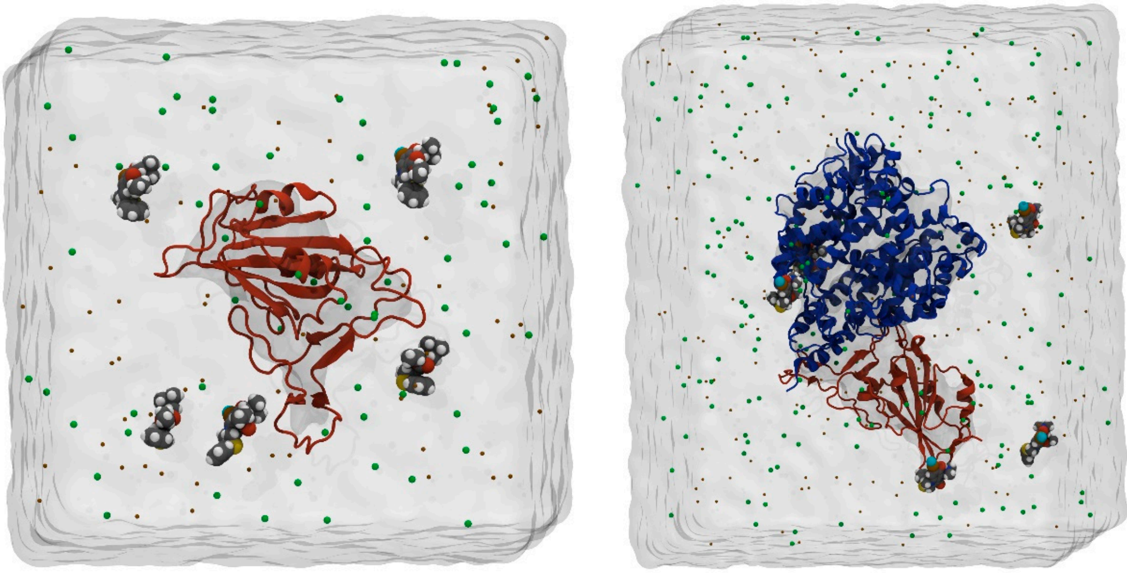

Figure-S2 RBD-5×Arb and ACE-2-RBD-5×Arb

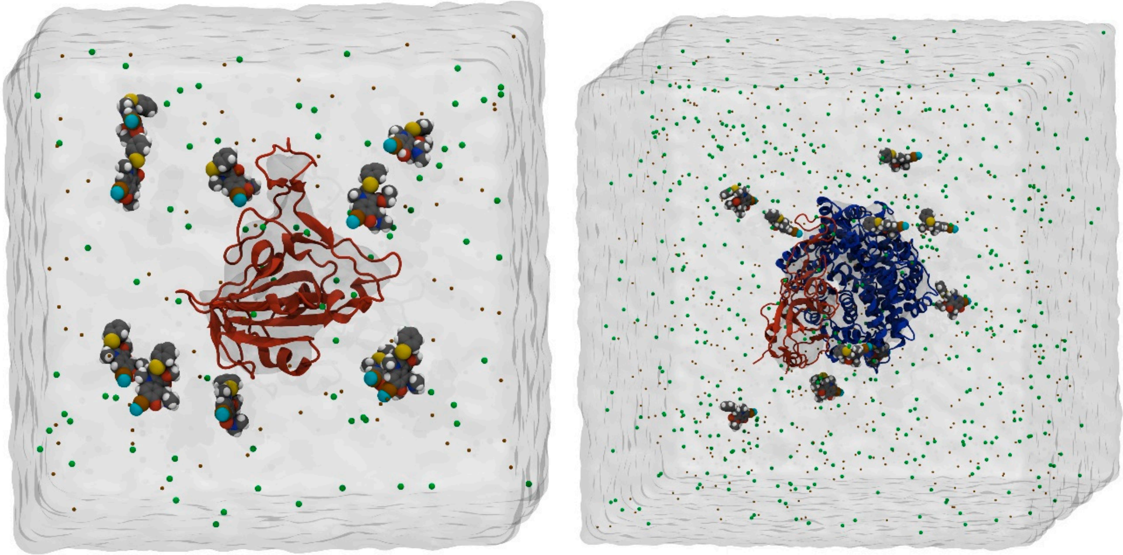

Figure-S3 RBD-10×Arb and ACE-2-RBD-10×Arb

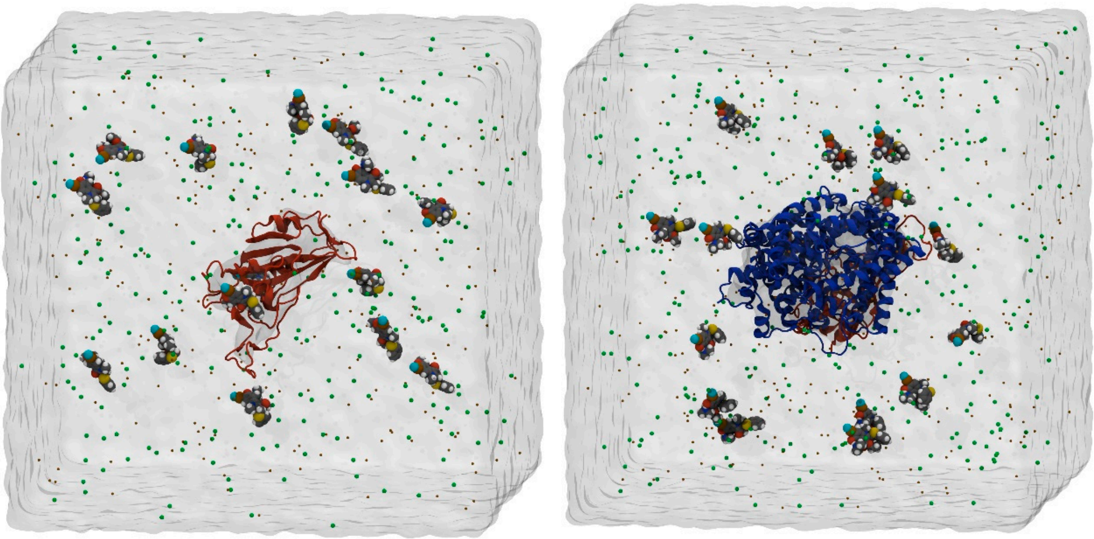

Figure-S4 RBD-15×Arb and ACE-2-RBD-15×Arb

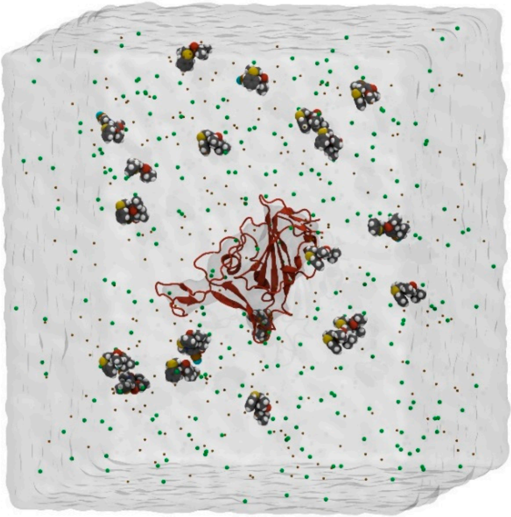
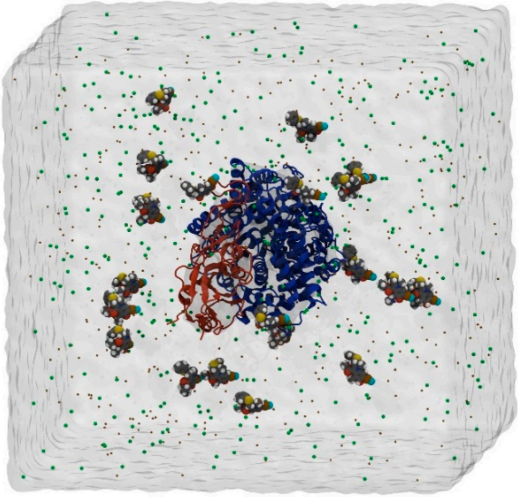

Figure-S5 RBD-20×Arb and ACE-2-RBD-20×Arb

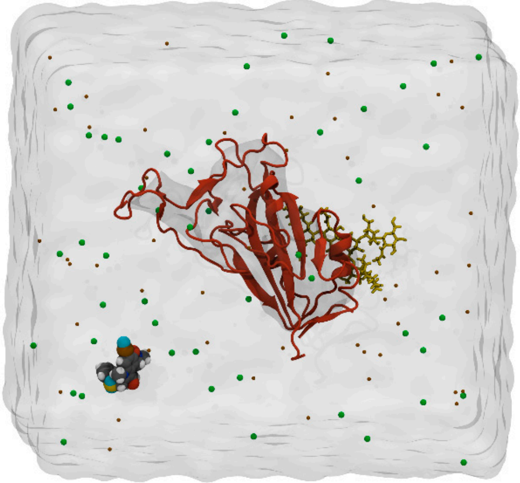
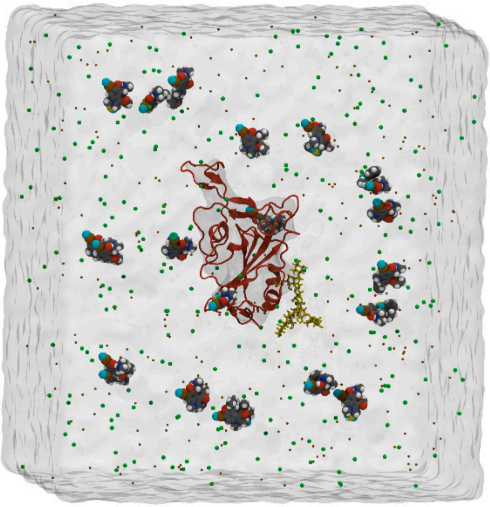

Figure-S6 RBD-20×Arb and ACE-2-RBD-20×Arb

RBD analysis results

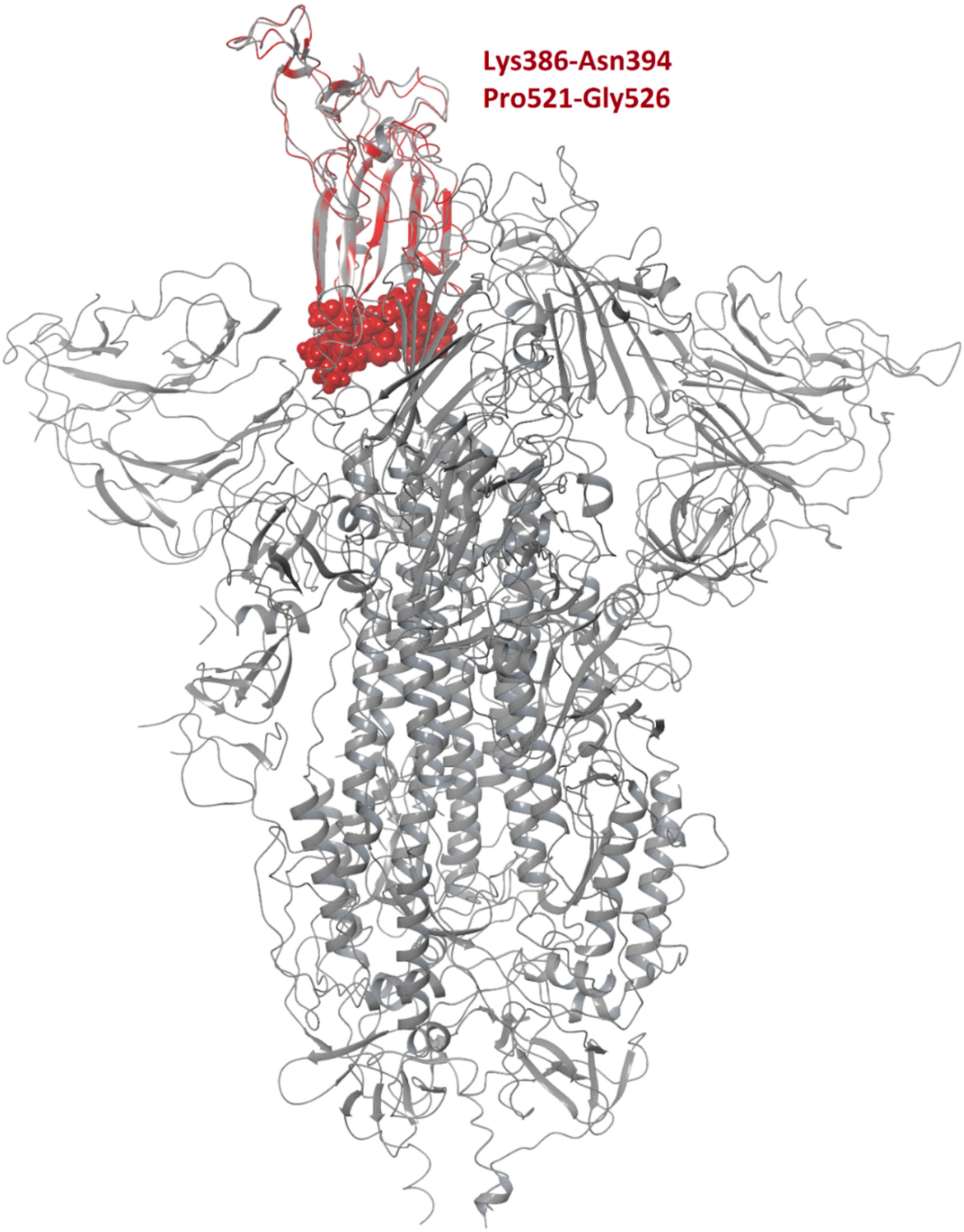

Figure-S7 Spike-protein SARS-CoV-2 open conformation: hard-to-reach amino acids for Arbidol are shown with balls model

Root-mean-square deviation of atomic positions

The results of molecular dynamics simulation for 300 ns at 310K are presented. In the case of ligands for systems containing 1 and 5 Arbidol molecules (RBD-n×Arb and ACE-2-RBD-n×Arb, n = 1, 5), perturbations in RMSD values are observed (Figures S 8-9), which fade with increasing numbers of Arbidol molecules (RBD-n×Arb and ACE-2-RBD-n×Arb, n = 10-20) (Figures S 10-12). At the same time, increasing the simulation time at an elevated temperature does not practically change the situation. The perturbations of the RMSD values of the ligand position in the RBD-1×Arb and ACE-2-RBD-1×Arb systems are significant (Figure S 13), while in RBD-20×Arb and ACE-2-RBD-20×Arb they are not significant (Figure S 14).

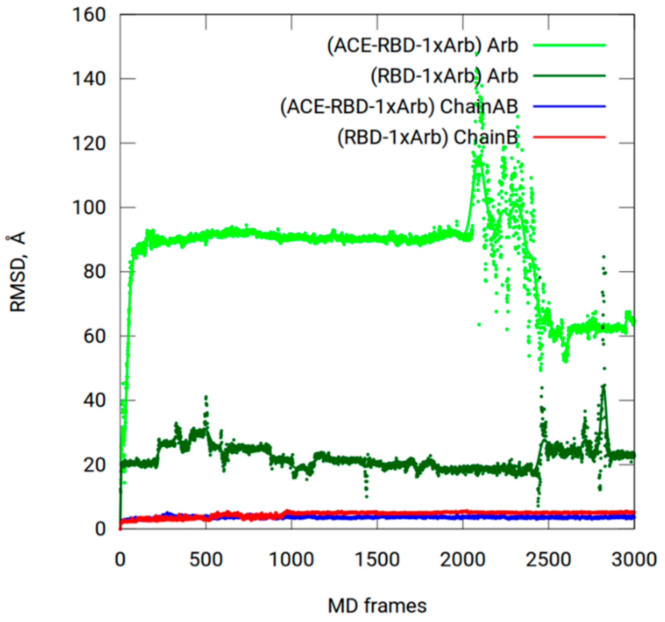

Figure-S8 RMSD of ACE-2-RBD – 1×Arb molecular dynamic simulation

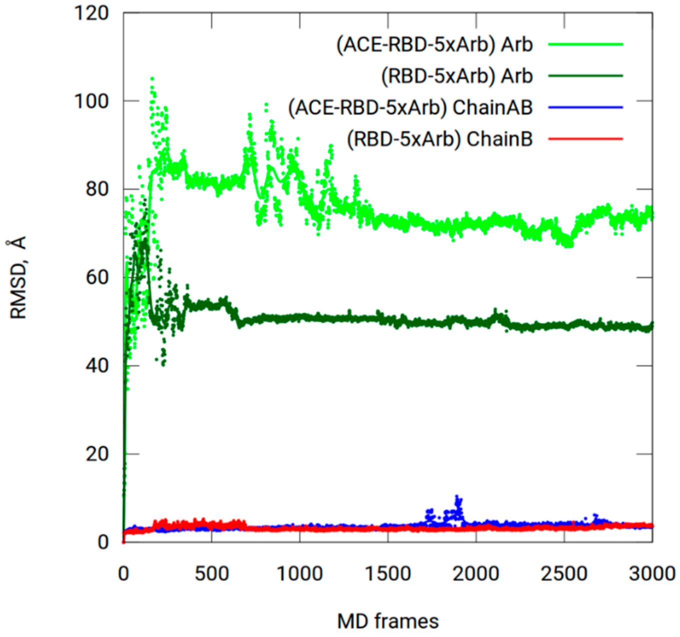

Figure-S9 RMSD of ACE-2-RBD – 5×Arb molecular dynamic simulation

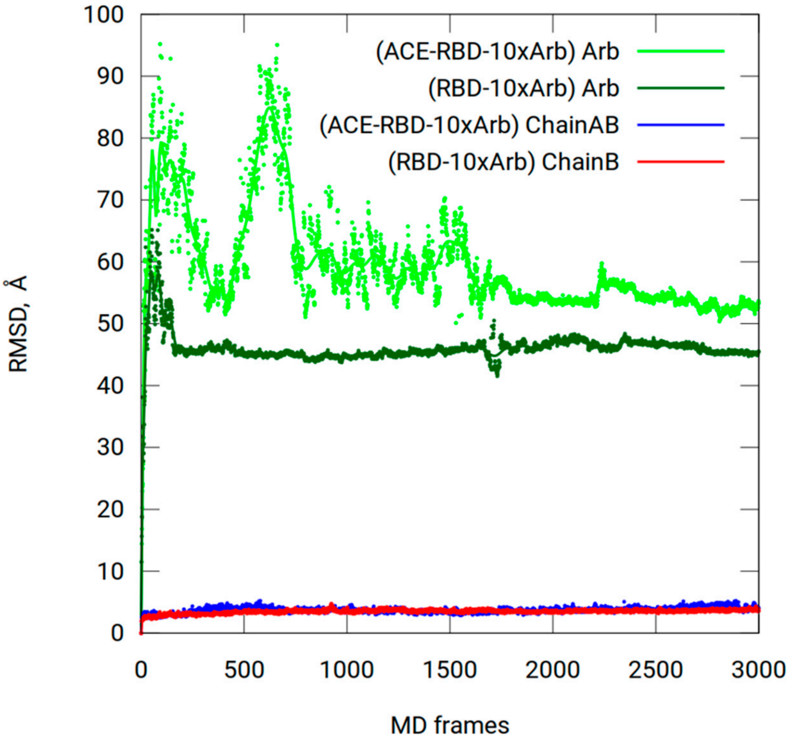

Figure-S10 RMSD of ACE-2-RBD – 10×Arb molecular dynamic simulation

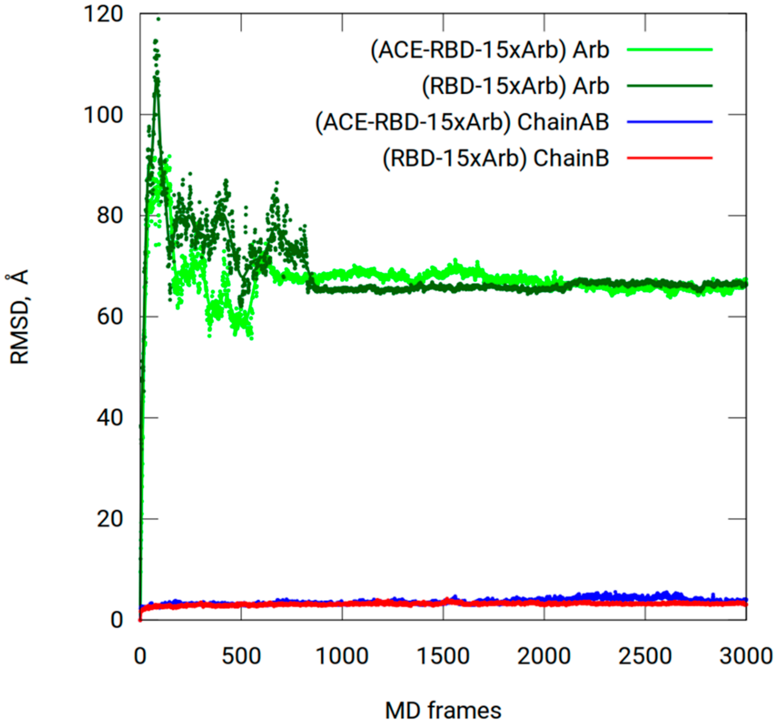

Figure-S11 RMSD of ACE-2-RBD – 15×Arb molecular dynamic simulation

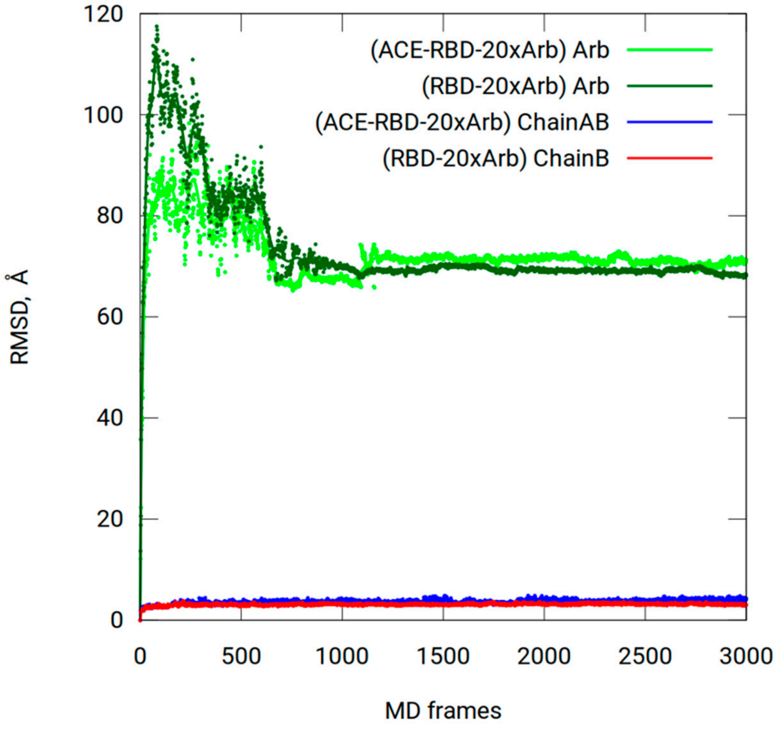

Figure-S12 RMSD of ACE-2-RBD – 20×Arb molecular dynamic simulation

The results of molecular dynamics simulation for 100 ns at 400K are presented.

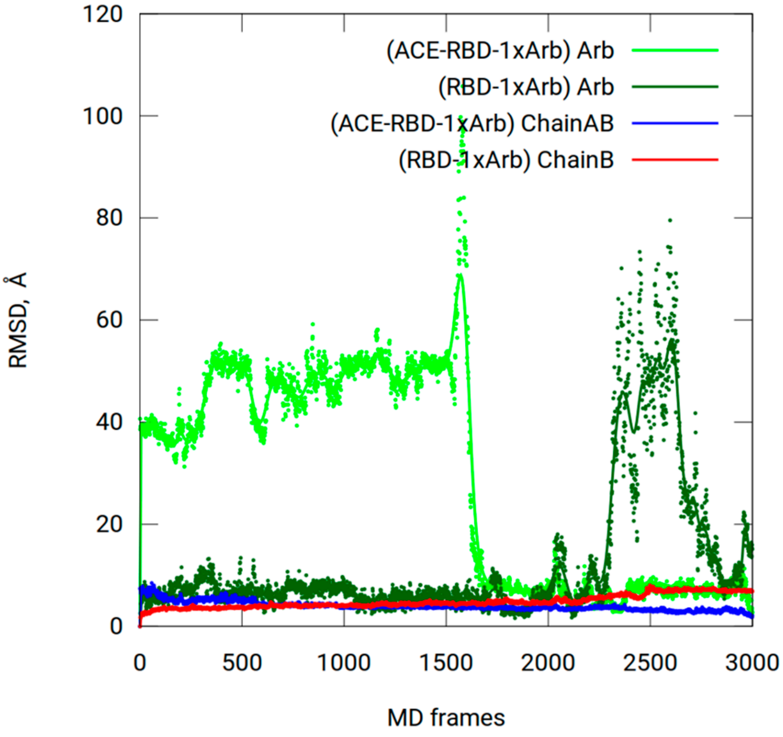

Figure-S13 RMSD of ACE-2-RBD – 1×Arb molecular dynamic simulation

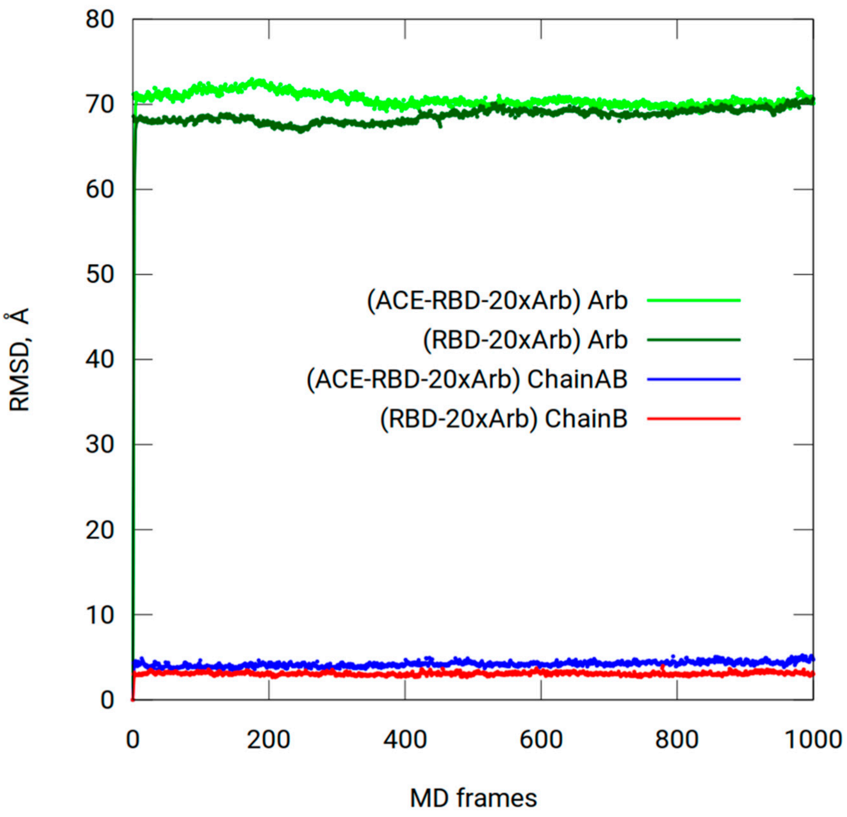

Figure-S14 RMSD of ACE-2-RBD – 20×Arb molecular dynamic simulation

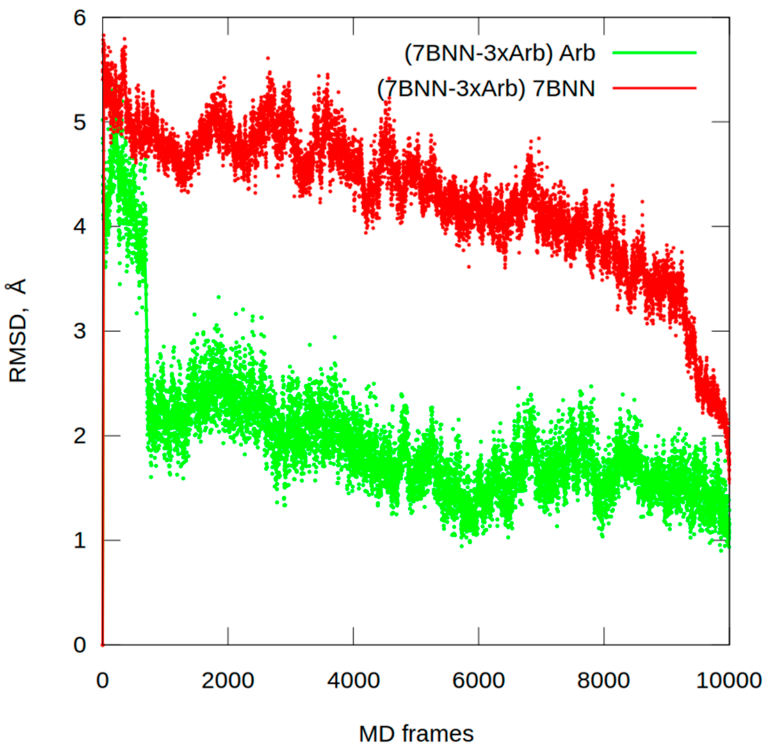

Figure-S15 RMSD of S-protein 3×Arb molecular dynamic simulation

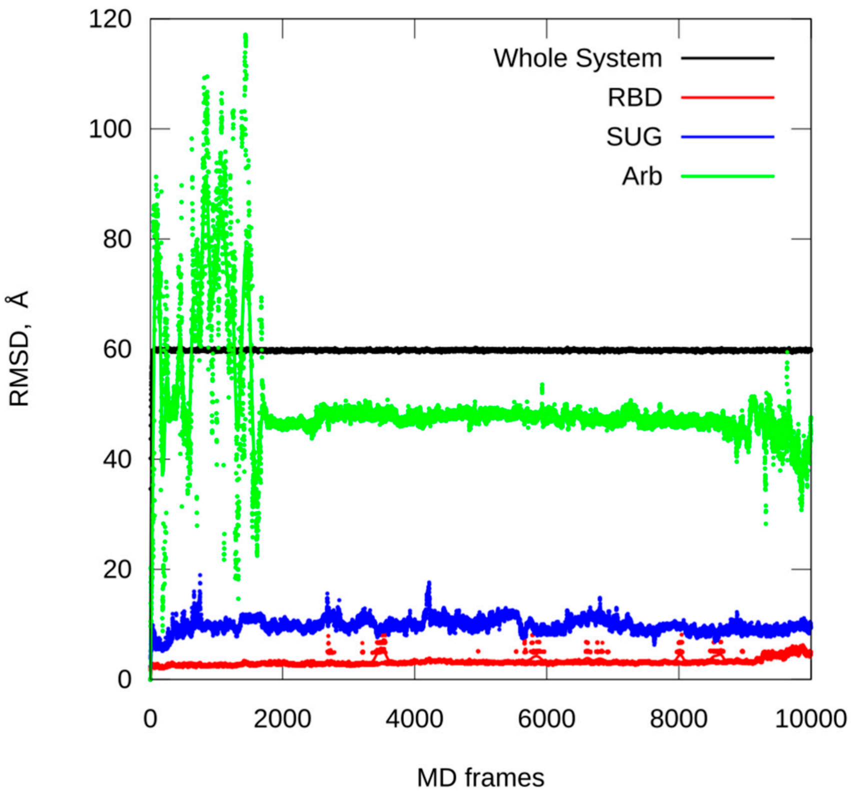

Figure-S16 RMSD of RBD-SUR-1×Arb molecular dynamic simulation

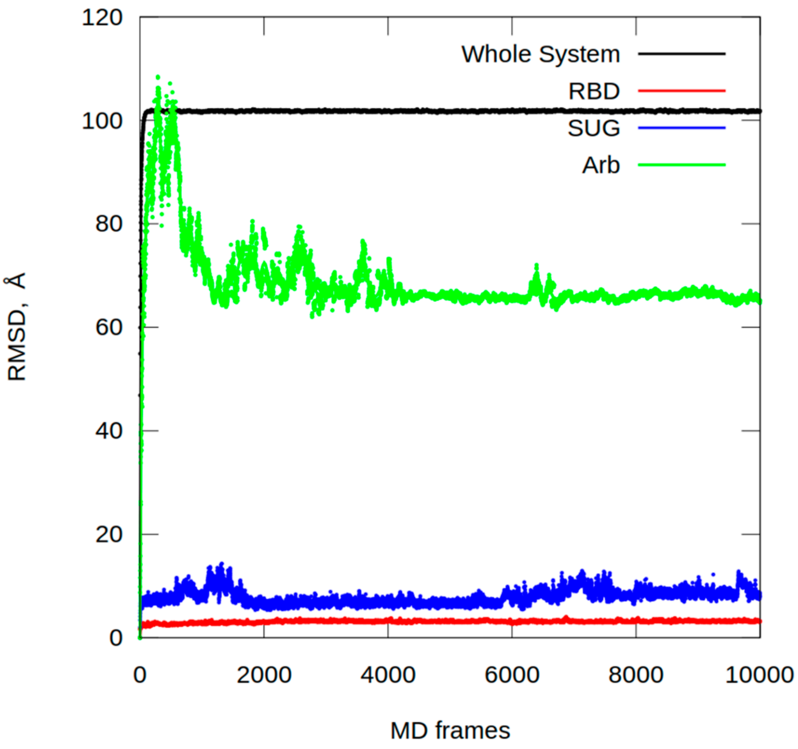

Figure-S17 RMSD of RBD-SUR-20×Arb molecular dynamic simulation

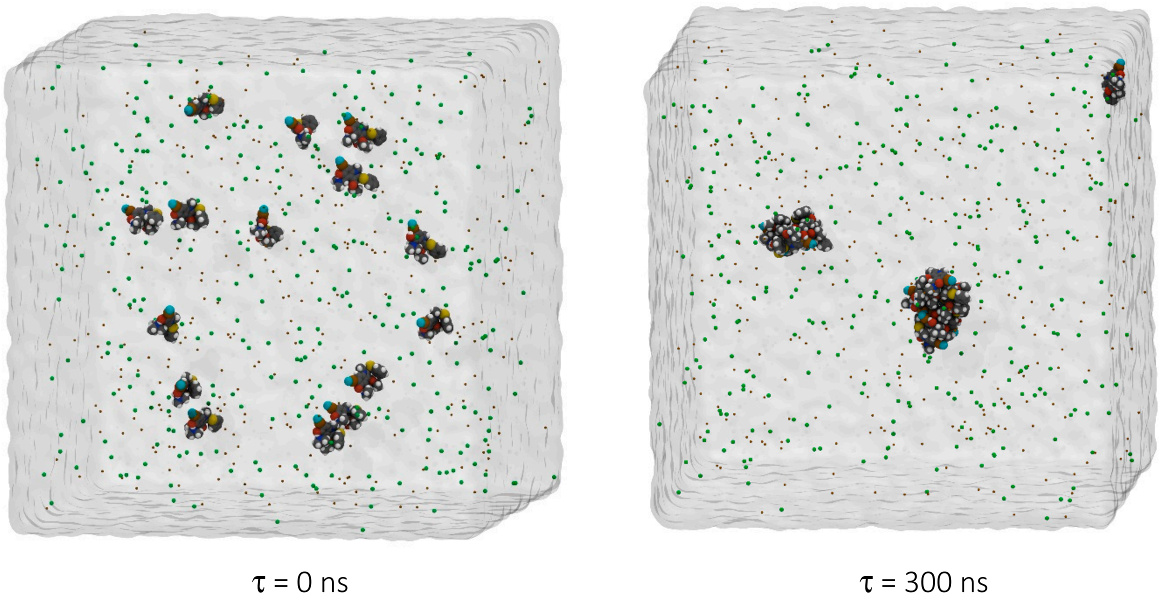

Figure-S18

Population analysis

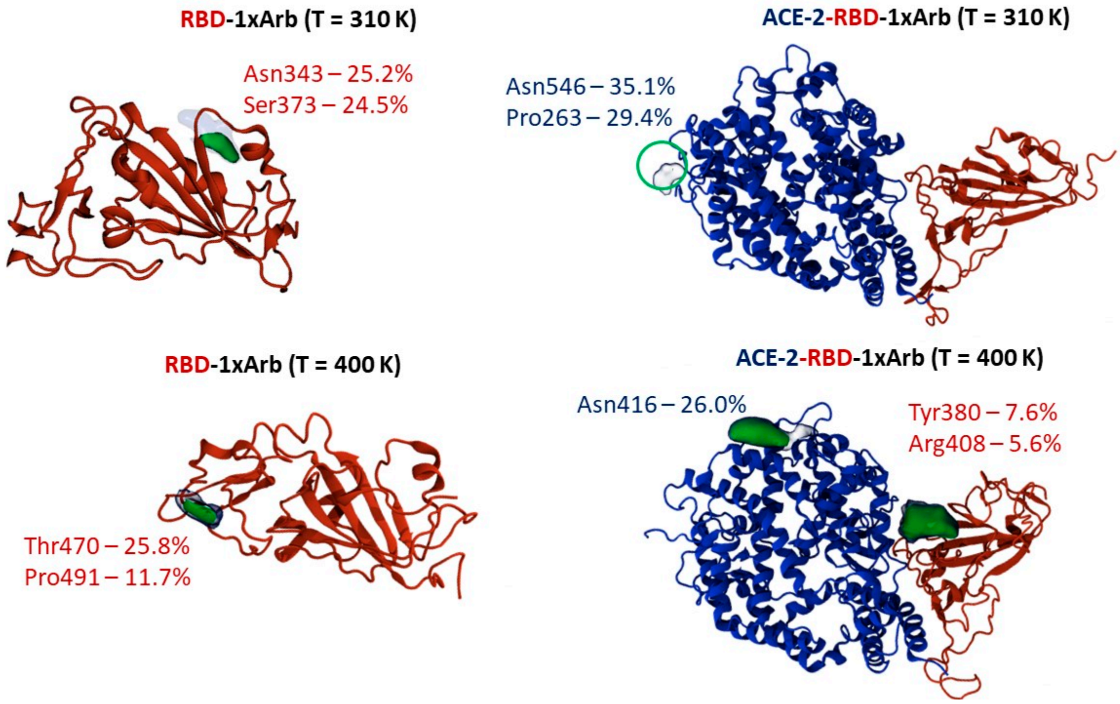

Figure-S19

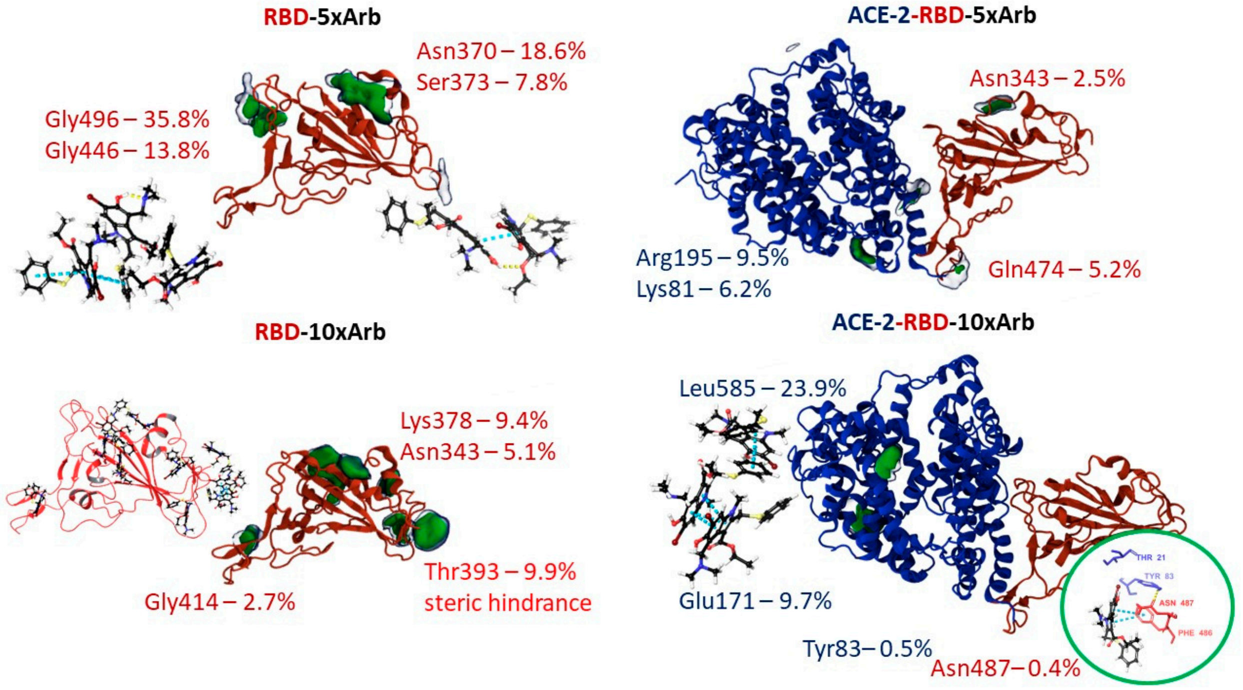

Figure-S 20

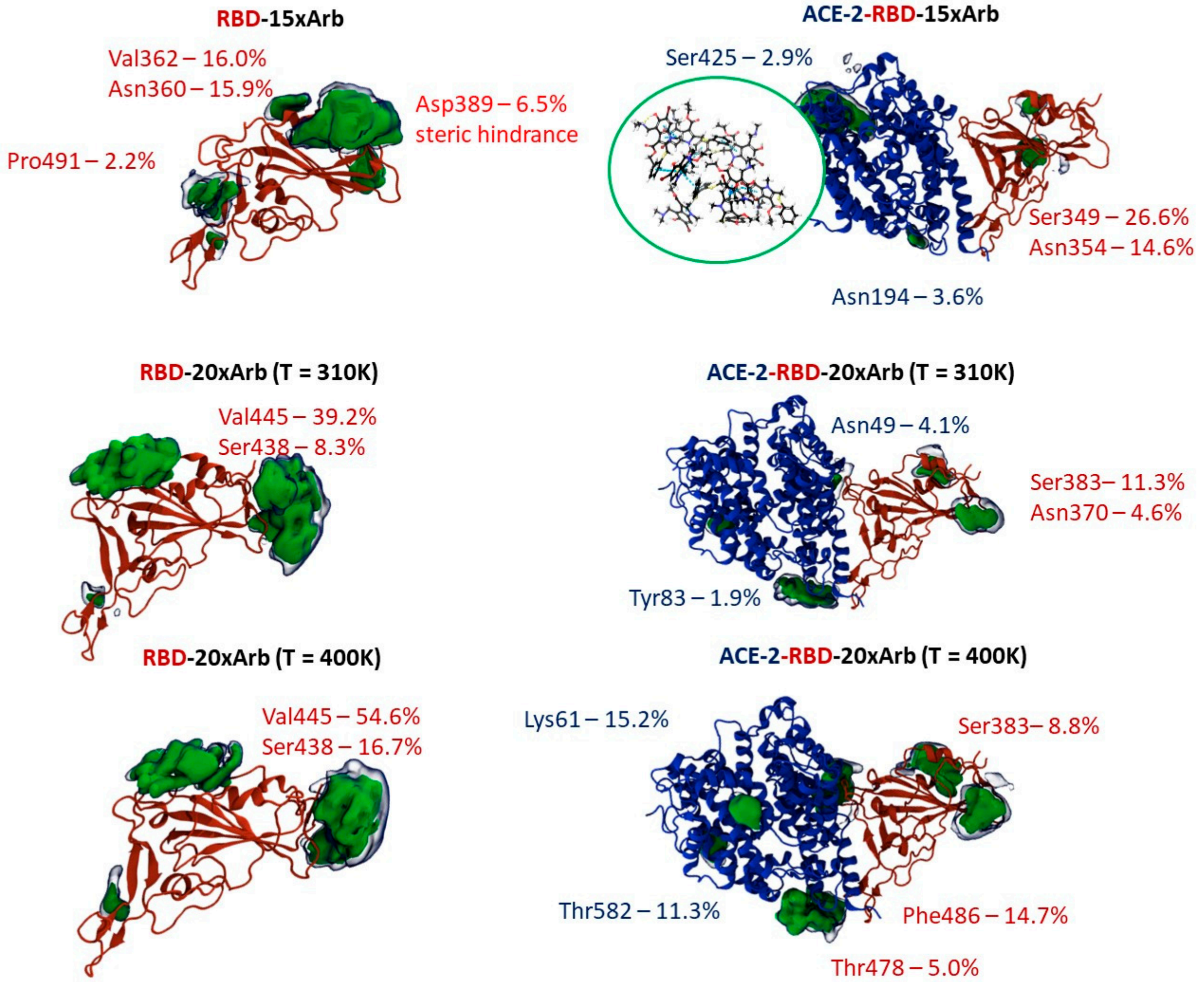

Figure-S21

Table S1 Molecular dynamics statistics for systems RBD-n×Arb, n = 1..20 and RBD-SUG-n×Arb, (n = 1, 20)

System	Amino acids	Donor-acceptor interaction	Descript of Arb	Occupancy, %	Probability of intramolecular interaction, %	Type of intramolecular interaction
RBD-1×Arb (310 K)	Asn343	Donor	O – acceptor	0.8	25.5	H-bond
	Ser371	Donor	O – acceptor	0.8	24.5	H-bond
	Asn370	Donor	O – acceptor	0.4	12.6	H-bond
RBD-1×Arb (400 K)	Thr470	Donor	O – acceptor	0.8	27.6	H-bond
	Pro491	Donor	Ring	0.3	10.3	π-cation
	Arg545	Donor	O – acceptor	0.3	10.3	H-bond
RBD-SUG-1×Arb (310 K)	Pro491	Donor	Acceptor	0.4	19.8	hydrophobic
	Tyr489	Donor	Acceptor	0.4	14.0	hydrophobic
	Tyr473	Donor	Acceptor	0.2	9.0	hydrophobic
RBD-5×Arb (310 K)	**Gly496**	Donor	O – acceptor	**25.4**	35.6	H-bond
	**Asn370**	Donor	O – acceptor	**13.2**	18.6	H-bond
	Gly446	Donor	O – acceptor	9.8	13.4	H-bond
RBD-10×Arb (310 K)	Thr393	Donor	Ring	3.7	9.9	π-cation
	Lys378	Donor	N - acceptor	3.5	9.4	H-bond
	Asn343	Acceptor	O - donor	1.8	5.1	H-bond
RBD-15×Arb (310 K)	**Val362**	Donor	Ring	6.3	16.0	π-cation
	**Asn360**	Donor	O - acceptor	6.3	15.9	H-bond
	Asp389	Donor	O - acceptor	2.6	6.5	H-bond
RBD-20×Arb (310 K)	**Val445**	Donor	O - acceptor	**31.3**	54.6	H-bond
	**Ser438**	Donor	Ring	9.6	16.7	π-cation
	Asn388	Acceptor	O - donor	2.6	4.5	H-bond
RBD-SUG-20×Arb (310 K)	Gly381	Donor	Acceptor	12.5	21.6	H-bond
	Gly339	Donor	Acceptor	7.4	12.5	hydrophobic
	Asn440	Donor	Acceptor	6.7	8.3	hydrophobic
RBD-20×Arb (400 K)	Val445	Donor	O - acceptor	29.3	39.2	H-bond
	Ser438	Donor	Ring	6.2	8.3	π-cation
	Gln93	Donor	O - acceptor	3.9	5.2	H-bond

Table S2 Molecular dynamics statistics for systems ACE-2-RBD-n×Arb, n = 1…20.

System	Amino acids	Donor-acceptor interaction	Descript of Arb	Occupancy, %	Probability of intramolecular interaction, %	Type of intramolecular interaction
ACE-2-RBD-1×Arb (310 K)	Asn456 (ACE-2)	Donor	O – acceptor	1.8	35.1	H-bond
	Pro263 (ACE-2)	Donor	Ring	1.4	29.4	π-cation
	Ala614 (ACE-2)	Donor	O – acceptor	0.6	12.0	H-bond
ACE-2-RBD-1×Arb (400 K)	Asn456 (ACE-2)	Donor	O – acceptor	1.7	26.0	H-bond
	Tyr380 (RBD)	Donor	O – acceptor	0.4	7.6	H-bond
	Pro590 (ACE-2)	Donor	Ring	0.3	5.6	π-cation
ACE-2-RBD-5×Arb (310 K)	Leu585 (ACE-2)	Acceptor	O - donor	5.9	23.9	H-bond
	Glu171 (ACE-2)	Acceptor	O - donor	2.4	9.7	H-bond
	Arg115 (Ace-2)	Donor	N - acceptor	1.4	5.5	H-bond
ACE-2-RBD-10×Arb (310 K)	Asn195 (ACE-2)	Donor	O – acceptor	6.0	9.5	H-bond
	Lys81 (ACE-2)	Donor	N - acceptor	3.9	6.2	H-bond
	Gln474 (RBD)	Donor	N - acceptor	3.3	5.3	H-bond
ACE-2-RBD-15×Arb (310 K)	Ser349 (RBD)	Donor	O – acceptor	18.5	26.6	H-bond
	Asn354 (RBD)	Donor	O – acceptor	10.2	14.6	H-bond
	Ser371 (RBD)	Donor	O – acceptor	2.9	4.3	H-bond
ACE-2-RBD-20×Arb (310 K)	Ser383 (RBD)	Donor	O – acceptor	11.1	11.6	H-bond
	Ala522 (RBD)	Donor	O – acceptor	8.2	8.5	H-bond
	Thr393 (RBD)	Donor	Ring	6.4	6.7	π-cation
ACE-2-RBD-20×Arb (400K)	Lys61 (ACE-2)	Donor	N – acceptor	13.0	15.2	H-bond
	Phe486 (RBD)	Donor	O – acceptor	12.6	14.7	H-bond
	Thr582 (ACE-2)	Donor	O – acceptor	9.6	11.3	H-bond

Table S3 Molecular dynamics statistics for systems Spike-protein-3×Arb

Amino acids	Descript of Arb	Occupancy, %	Probability of intramolecular interaction, %	Type of intramolecular interaction
Phe1042	hydrophobic	19.4	44.5	hydrophobic
Arg1019	O – donor	15.8	36.4	H-bond
Asn1023	N – acceptor	3.3	7.5	H-bond
Lys1028	O – donor	1.5	3.4	H-bond

Molecular docking results

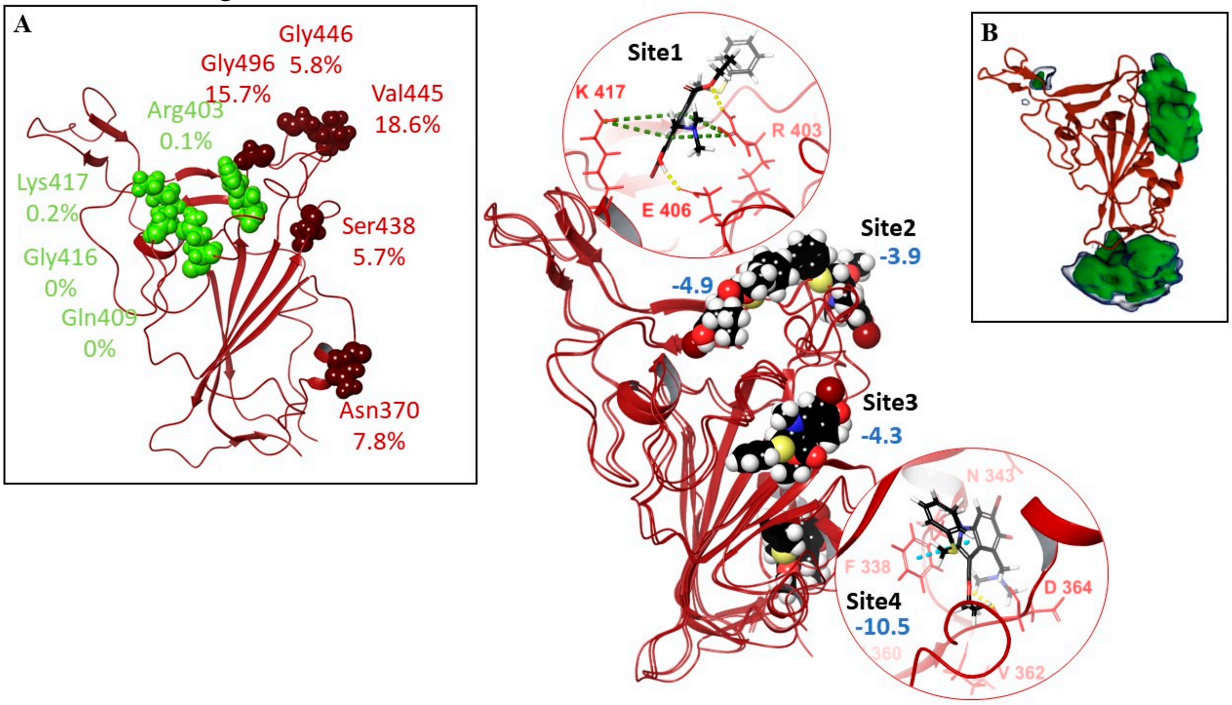

Figure-S22 Molecular docking results to potential RBD binding sites: Numbers show glide score values (kcal/mol). Hydrogen and salt bridges are shown with yellow and purple dashed lines, π-cationic and π-π stacking interactions with green and blue lines, respectively. A - Amino acid residues with which Arbidol molecules are most likely to interact are shown in dark red; light green amino acid residues are described in [26] as the likely binding site of Arbidol. B Weighted populations of Arbidol molecules on the protein surface because of statistical processing of molecular dynamic simulation data of the RBD-20×Arb system are shown.

Table S4 Docking results into the sites of PBD

Binding site	Glide score	|LE|	Glide Emodel	IFS score	Interaction	
					H-bond	Others
Site 1	-4.94	0.17	-56.76	-379.01	Arg403 Glu406	Lys417 Arg403 π-cation
Site 2	-3.88	0.13	-42.53	-387.31	Val445	Asn439 – salt bridge
Site 3	-4.33	0.15	-47.60	-387.99		
Site 4	-10.48	0.36	-88.75	-397.31	Asp364	Phe338 π-π
Site 4* (RBD-SUG)	-9.94	0.34	-84.76	-411.04	NAG	Phe373 π-cation Phe374 π-π

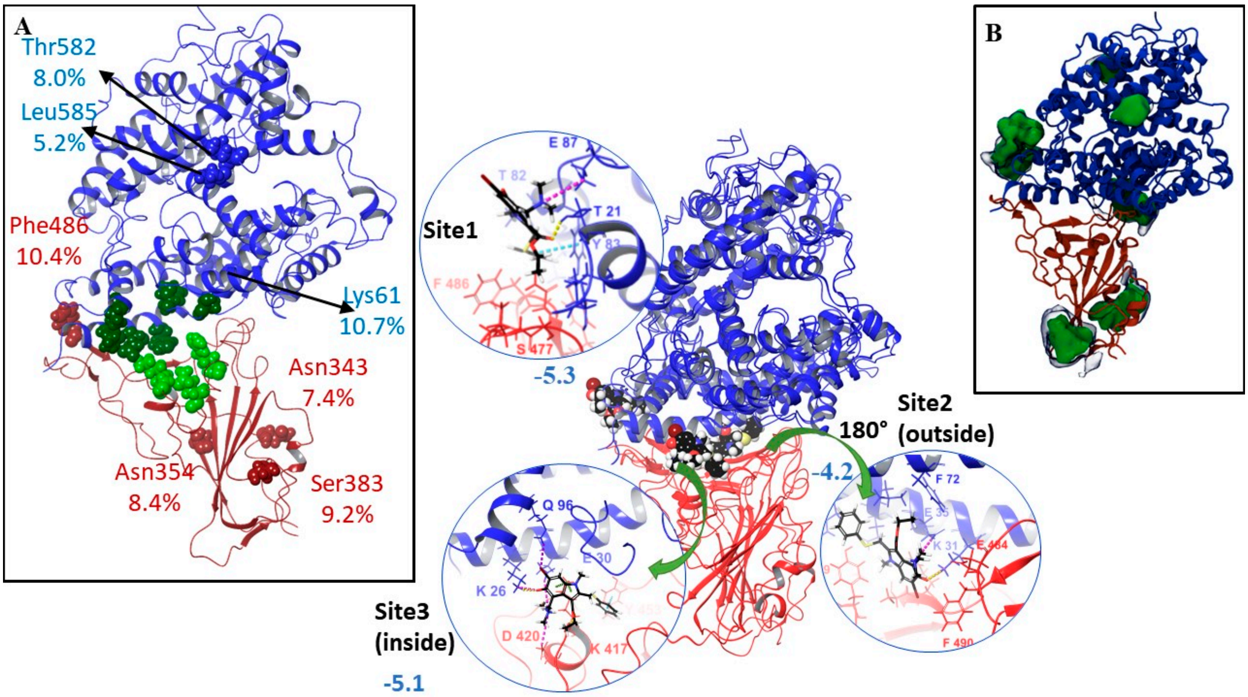

Figure-S23 ACE-2-RBD-20 Arb. Molecular docking results to potential RBD binding sites. Numbers show glides score values. Hydrogen bonds and salt bridges are shown in yellow and purple dashed lines π-cationic and π-π-stacking interactions in green and blue lines, respectively. A amino acid residues with which Arbidol molecules are most likely to interact are shown in dark red; green amino acid residues are described in 26 as the likely binding site of Arbidol (dark green amino acids refer to ACE-2; light green to RBD). B - Weighted average of Arbidol molecules on the protein surface resulting from statistical processing of data from molecular dynamic simulations of the ACE-2-RBD-20×Arb system are shown.

Table S5 Docking results into the interface of RBD-ACE-2 complex

Binding site	Glide score	|LE|	Glide Emodel	IFD score	Interaction	
					H-bond	Others
Site 1	-5.28	0.18	-50.25	-1690.20	ACE-2: Thr21	ACE-2: Glu-87 salt Tyr83 - π-cation
Site 2 (outside)	-4.20	0.15	-46.62	-1684.09	ACE-2: Lys31	ACE-2: Lys31, Glu35
Site 3 (inside)	-5.11	0.17	-52.72	-1689.87	ACE-2: Lys26 RBD: Lys417	ACE-2: Gln96 halogen bond Lys26, Glu30 salt RBD: Lys417 π-cation Tyr453 π-π

Table S6 Docking results into the subunits2

Binding site	Glide score	|LE|	Glide Emodel	IFD score	Interaction	
					H-bond	Others
Subunits 2 (FP2)	-7.70	0.27	-67.63	-3605.20	Arg1019	Glu-780 salt Leu727, Val1040, Phe1042, Leu1024 hydrophobic
